# Genome-wide studies reveal the essential and opposite roles of ARID1A in controlling human cardiogenesis and neurogenesis from pluripotent stem cells

**DOI:** 10.1186/s13059-020-02082-4

**Published:** 2020-07-09

**Authors:** Juli Liu, Sheng Liu, Hongyu Gao, Lei Han, Xiaona Chu, Yi Sheng, Weinian Shou, Yue Wang, Yunlong Liu, Jun Wan, Lei Yang

**Affiliations:** 1 Department of Pediatrics, Herman B Wells Center for Pediatric Research, Indiana University School of Medicine, 1044 W Walnut Street, R4 272, Indianapolis, IN 46202 USA; 2 Department of Medical and Molecular Genetics, Indiana University School of Medicine, Indianapolis, IN 46202 USA; 3 Department of Obstetrics, Gynecology & Reproductive Sciences, Magee-Women’s Research Institute, University of Pittsburgh, Pittsburgh, PA 15213 USA; 4 Center for Computational Biology and Bioinformatics, Indiana University School of Medicine, Indianapolis, IN 46202 USA; 5 Department of BioHealth Informatics, Indiana University School of Informatics and Computing, Indiana University – Purdue University Indianapolis, Indianapolis, IN 46202 USA

**Keywords:** SWI/SNF, Chromatin remodeling, ARID1A, REST, Cardiogenesis, Neurogenesis, Pluripotent stem cells

## Abstract

**Background:**

Early human heart and brain development simultaneously occur during embryogenesis. Notably, in human newborns, congenital heart defects strongly associate with neurodevelopmental abnormalities, suggesting a common gene or complex underlying both cardiogenesis and neurogenesis. However, due to lack of in vivo studies, the molecular mechanisms that govern both early human heart and brain development remain elusive.

**Results:**

Here, we report ARID1A, a DNA-binding subunit of the SWI/SNF epigenetic complex, controls both neurogenesis and cardiogenesis from human embryonic stem cells (hESCs) through distinct mechanisms. Knockout-of-ARID1A (ARID1A^−/−^) leads to spontaneous differentiation of neural cells together with globally enhanced expression of neurogenic genes in undifferentiated hESCs. Additionally, when compared with WT hESCs, cardiac differentiation from ARID1A ^−/−^ hESCs is prominently suppressed, whereas neural differentiation is significantly promoted. Whole genome-wide scRNA-seq, ATAC-seq, and ChIP-seq analyses reveal that ARID1A is required to open chromatin accessibility on promoters of essential cardiogenic genes, and temporally associated with key cardiogenic transcriptional factors T and MEF2C during early cardiac development. However, during early neural development, transcription of most essential neurogenic genes is dependent on ARID1A, which can interact with a known neural restrictive silencer factor REST/NRSF.

**Conclusions:**

We uncover the opposite roles by ARID1A to govern both early cardiac and neural development from pluripotent stem cells. Global chromatin accessibility on cardiogenic genes is dependent on ARID1A, whereas transcriptional activity of neurogenic genes is under control by ARID1A, possibly through ARID1A-REST/NRSF interaction.

## Introduction

Human cardiogenesis and neurogenesis occur contemporaneously, and both have a similar process of morphogenesis including germ layer segregation, progenitor cell differentiation, cell fate specification, cell migration, left/right patterning, and dorsal/ventral patterning [1–3]. Heart and brain development also share complex cellular interactions. For example, neural crest cells (NCCs), which are capable of differentiation into peripheral neurons and glia, are also able to migrate from the pharyngeal arch arteries (PAA) and heart outflow tract (OFT) to the primitive heart and give rise to smooth muscle, connective tissue, and great arteries of the heart [4]. Notably, newborns with congenital heart defects exhibited a high frequency of neurodevelopmental deficits [4, 5]. Previous studies revealed that epigenetic regulatory mechanisms, such as DNA methylation [6], histone modifications [7], and chromatin remodeling [8–11], played essential roles in heart and neural development in mammals. However, the molecular mechanisms driving both cardiogenesis and neurogenesis in human embryos remain unclear. A better understanding of these mechanisms is critical for studying etiology of human congenital cardiac and neural defects.

The evolutionarily conserved ATP-dependent SWI/SNF complex is one of the largest chromatin-remodeling complexes, consisting of as many as 15 subunits, including SMARCA2 (also known as BRM) or SMARCA4 (also known as BRG1) as the ATPase catalytic subunit [12]. Several BRG1-associated factors (BAFs), such as ARID1A (Baf250a), have DNA binding capacity and assemble with either BRM or BRG1 to form a functional chromatin-remodeling complex [13]. BAFs also associate with other co-factors to determine the identity of a given SWI/SNF chromatin-remodeling complex and which consequently dictate where that complex will act [14]. As previously reported, ARID1A (Baf250a) binds promoter regions of transcription factors in a sequence-specific manner to drive SWI/SNF recruitment [15]. Loss of Ariad1a in mice results in embryonic lethality and developmental arrest at around day E6.5, showing lack of primitive streak or mesoderm formation [16]. A single amino acid mutation (Arid1aV^1068G/V1068G^) impaired Arid1a-DNA interactions [15] and resulted in neural defects including cranioschisis and neural tube defects, as well as cardiac defects including defective trabeculation, hypoplastic myocardial walls, and ventricular septal malformation. Additionally, homozygous loss-of-ARID1a in mouse neural crest cells resulted in embryonic lethality at around E15, with prominent heart defects that included incomplete formation of the cardiac outflow tract septum and defective posterior pharyngeal arteries [17]. ARID subunits are among the most frequently mutated SWI/SNF subunits found in human disease [17]. Mutations in 4 different SWI/SNF subunits including ARID1A/B were identified in three congenital syndromes that include both neural and cardiac defects: Coffin-Siris syndrome (CSS), Nicolaides-Baraitser syndrome (NCBRS), and ARID1B-related intellectual disability (ID) syndrome [13, 18–21]. Patients with these syndromes show severe intellectual deficits as well as cardiac defects such as atrial/ventricular septal defects, patent ductus arteriosus (PDA), mitral and pulmonary atresia, aortic stenosis, and single right ventricle. These data indicate that abnormal ARID1A activity can lead to defective formation of both the heart and brain in humans. However, the molecular mechanisms by which ARID1A controls human cardiogenesis and neurogenesis still remain elusive.

In this study, we investigate the roles of ARID1A in early human cardiac and neural development by using an in vitro human embryonic stem cell (hESC) model [22–25]. Expression of ARID1A is upregulated during early cardiac differentiation from hESCs. Surprisingly, knockout-of-ARID1A in hESCs (ARID1A^−/−^) led to spontaneous neural differentiation even under pluripotent stem cell culture conditions. Additionally, under conditions of targeted cardiac differentiation, ARID1A^−/−^ hESCs gave rise to robustly increased numbers of neural cells, including neural stem cells and neurons, whereas cardiac differentiation was significantly suppressed when compared with WT hESCs. scRNA-seq revealed cellular and transcriptional heterogeneities between ARID1A^−/−^ and WT hESCs. ATAC-seq (Assay for Transposase-Accessible Chromatin using sequencing) demonstrated changes in chromatin accessibility on ARID1A-occupied cardiogenic and neurogenic genes in ARID1A^−/−^ vs. WT hESCs, while ChIP-seq revealed differences in genome-wide ARID1A occupancy on promoters of essential cardiogenic and neurogenic genes. When integrated, these unbiased genome-wide studies revealed loss-of-ARID1A globally reduced chromatin accessibility and expression of essential cardiogenic genes, but did not uniformly affect chromatin accessibility on neurogenic genes. Furthermore, we performed Co-IP to show ARID1A interacted with key cardiac transcriptional factors T and MEF2C at the mesoderm and cardiac progenitor cell formation stages during human cardiogenesis. We identified that ARID1A recruited the transcriptional repressor element-1 (RE1) silencing transcription factor/neuron-restrictive silencer factor (REST/NRSF) to co-occupy the promoters of neurogenic genes, but not cardiogenic genes, thereby suppressing transcription of neurogenic genes. Collectively, our findings reveal distinct and novel mechanisms by which ARID1A drives early human cardiogenesis and neurogenesis, primarily via chromatin remodeling and gene transcription regulation, respectively.

## Results

### Whole mRNA-seq predicts ARID1A in cardiac development from hESCs

**Fig. 1:**
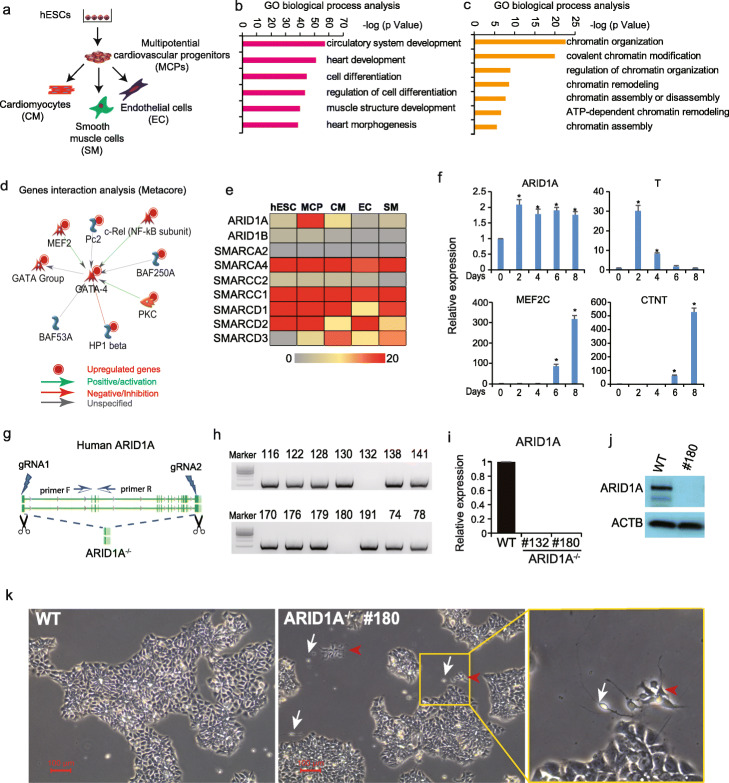
Loss-of-ARID1A induces spontaneous differentiation of hESCs. **a** Scheme for whole transcriptome sequencing of cardiovascular cell lineages derived from human embryonic stem cells (hESCs). **b**, **c** Gene Ontology (GO) biological process analysis of upregulated genes (MCP vs. hESCs) showing enriched heart development events (**b**) and epigenetic regulation events (**c**) during human cardiac development. **d** Interaction network analysis of SWI/SNF complex subunits and other cardiogenic genes by Metacore software. **e** Gene expression profiles of SWI/SNF subunits in different cardiovascular lineages by whole transcriptome sequencing. **f** Expression dynamics of ARID1A and key cardiac genes during cardiac differentiation of H9 hESCs. RNAs were collected every 2 days from day 0 to day 10 of cardiac differentiation. **g** Strategy for completely knocking out human ARID1A in H9 hESCs using CRISPR/Cas9. **h** PCR screening for ARID1A^−/−^ hESC clones. **i** ARID1A mRNA expression detected by qRT-PCR. All bars are shown as mean ± SD. *n* = 3, *p < 0.05 (an unpaired two-tailed *t* test with Welch’s correction). **j** ARID1A protein expression levels detected by Western blot. **k** WT and ARID1A^−/−^ hESCs were cultured in mTesR medium. ARID1A^−/−^ hESCs displayed small clusters of differentiated cells (red arrow heads and white arrows indicate two different cell types). All functional analysis and gene interaction network analyses were done by Metacore (Clarivate Analytics)

Previously, we established a cardiovascular differentiation protocol from human embryonic stem cells (hESCs) by adding different combinations of growth factors including BMP4/bFGF/Activin A and VEGF/DKK1 at different stages [26]. Temporal analyses using whole mRNA sequencing analyses revealed that this cytokine treatment paradigm results in the progressive appearance of multipotential cardiovascular progenitors (MCPs), cardiomyocytes (CMs), smooth muscle cells (SMs), and endothelial cells (ECs) (Fig. 1a) [26, 27]. Gene Ontology (GO) biological process analysis by Metacore found the upregulated genes were significantly enriched into cardiac development (Fig. 1b) and chromatin organization events (Fig. 1c), suggesting a critical role for chromatin organization/remodeling mechanism(s) during early human cardiac development. Of interest, the upregulated genes included subunits of the SWI/SNF chromatin-remodeling complex, BAF250A (ARID1A) and BAF53A, which may regulate expression of GATA4 (a key cardiogenic transcription factor, Fig. 1d). This suggests a potential role of SWI/SNF chromatin-remodeling complex in early human heart development. Next, we profiled the expression pattern of essential SWI/SNF components in above five cell lineages by whole mRNA-seq (Fig. 1e). We found that expression level of ARID1A, a subunit of SWI/SNF complex with DNA binding capacity, significantly increased during hESC differentiation to cardiac progenitors (Fig. 1e). qRT-PCR analysis confirmed the increased expression of ARID1A during cardiac differentiation of hESCs (Fig. 1f). Cardiac troponin T (CTNT), which is a CM-specific marker gene, also showed increased expression during cardiac differentiation (Fig. 1f). Tissue-wide gene expression profiling demonstrated ARID1A in human adult heart tissue (Additional file 1: Fig. S1a). Given that loss-of-Arid1a in mouse leads to severe developmental defects in both the heart and neural tube [15], we decided to delete ARID1A expression in H9 hESCs by using dual gRNA-mediated CRISPR/Cas-9 technology (Fig. 1g, Additional file 1: S1b). Two ARID1A^−/−^ hESC clones (#132 and #180) were identified by PCR (Fig. 1h, Additional file 1: S1c). ARID1A mRNA (Fig. 1i) and protein (Fig. 1j) levels were undetectable in ARID1A^−/−^ clones when compared with WT hESCs. Large ARID1A^−/−^ hESC colonies displayed the same morphologies as WT hESCs. However, small cell clusters between large ARID1A^−/−^ colonies exhibited morphology of differentiated cell types (Fig. 1k, Additional file 1: S1d). Those differentiated cell types were not observed in WT hESCs, indicating loss-of-ARID1A may induce sporadic differentiation of hESCs.

### Single-cell RNA sequencing (scRNA-seq) reveals loss-of-ARID1A induces spontaneous neural differentiation

**Fig. 2:**
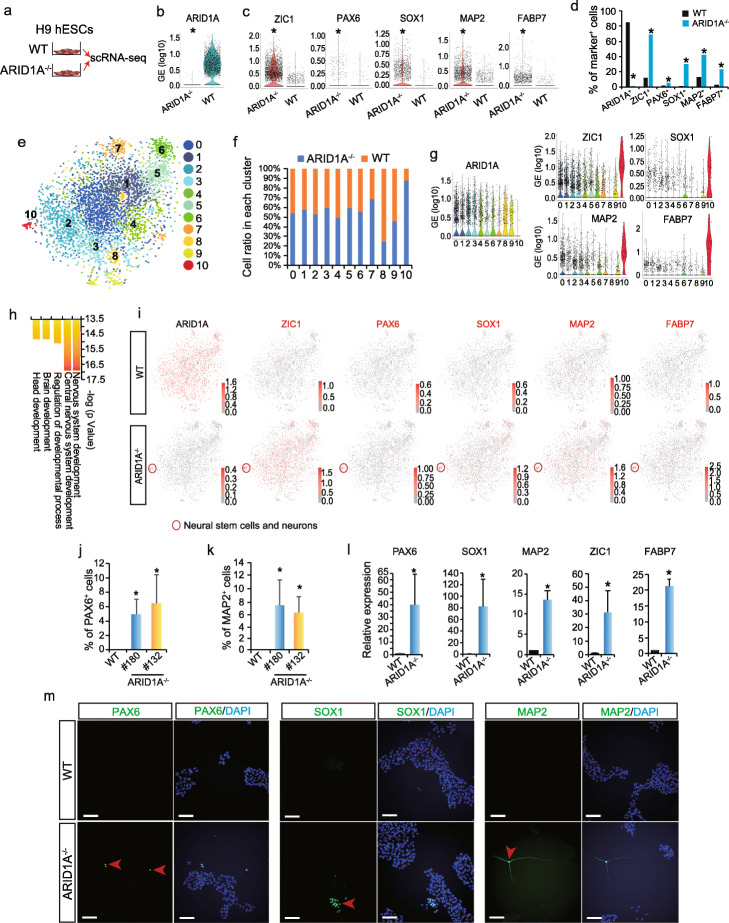
scRNA-seq reveals loss-of-ARID1A induces spontaneous neural differentiation in hESCs. **a** Undifferentiated WT and ARID1A^−/−^ hESCs were collected for single-cell RNA sequencing (scRNA-seq). **b** ARID1A expression in WT and ARID1A^−/−^ hESCs detected by scRNA-seq. *p < 2.4E−13 (Wilcoxon’s test). **c** Expression levels of neural stem cell markers (ZIC1, PAX6, SOX1) and neuron markers (MAP2, FABP7) in WT and ARID1A^−/−^ hESCs. *p < 2.4E−13 (Wilcoxon’s test). **d** Percentage of cells expressing ARID1A, neural stem cell markers (ZIC1, PAX6, SOX1), and neuron markers (MAP2, FABP7) in WT and ARID1A^−/−^ hESCs. *p < 1.1 E−14 (Fisher’s exact test). **e** Integrative analysis of scRNA-seq datasets from WT and ARID1A^−/−^ hESCs. Cell clusters were visualized with t-distributed stochastic neighbor embedding (t-SNE). **f** Ratios of WT and ARID1A^−/−^ hESCs in each cluster from integrative scRNA-seq data. **g** Violin plots of scRNA-seq data showing expression levels of ARID1A, neural stem cell markers (ZIC1, SOX1), and neuron markers (MAP2, FABP7) in each cluster. **h** GO biological process analysis for upregulated genes (ARID1A^−/−^ vs. WT) in cluster 10 by Metacore software. **i** Feature plots of scRNA-seq data showing the distribution of cells positively expressing ARID1A, neural stem cell markers (ZIC1, PAX6, SOX1), and neuron markers (MAP2, FABP7) in all clusters. Red circle indicates the cluster 10 containing neural stem cells and neurons solely derived from in ARID1A^−/−^ hESCs. **j**, **k** Flow cytometry data showing the percentage of PAX6^+^ (**j**) and MAP2^+^ (**k**) cells in WT and ARID1A^−/−^ hESCs. All bars are shown as mean ± SD. *n* = 3, *p < 0.05 (KO vs. WT, an unpaired two-tailed *t* test with Welch’s correction). **l** Expression levels of neural-associated markers analyzed by qRT-PCR. All bars are shown as mean ± SD. *n* = 3, *p < 0.05 (KO vs. WT, an unpaired two-tailed *t* test with Welch’s correction). **m** Immunomicroscopy to detect neural stem cell markers (PAX6, SOX1) and neuron marker (MAP2) in WT and ARID1A^−/−^ hESCs. Scale bar, 100 μm

To determine the identity of differentiated ARID1A^−/−^ hESCs, single-cell RNA sequencing (scRNA-seq) was performed (Fig. 2a). ARID1A mRNA was not detected in ARID1A^−/−^ hESCs, confirming global ARID1A deficiency (Fig. 2b). Interestingly, expressions of neural stem cell markers (ZIC1, PAX6, SOX1) and neuron markers (MAP2, FABP7) were all significantly increased in ARID1A^−/−^ compared with WT hESCs (*p* < 2.4E−13, Fig. 2c). Moreover, the percentage of cells positively expressing ZIC1, PAX6, SOX1, MAP2, and FABP7 were significantly higher in ARID1A^−/−^ hESCs than those in WT hESCs (*p* < 1.1E−14, Fig. 2d). In order to further compare the cellular heterogeneities, scRNA-seq data from both WT and ARID1A^−/−^ hESCs were integrated and then interrogated with Seurat (an R toolkit for single-cell genomics [28]), to perform dimension reduction and cell clustering. In total, 11 clusters were identified, as visualized by the t-distributed stochastic neighbor embedding (t-SNE) (Fig. 2e) [28, 29]. This revealed high heterogeneities of hESCs under pluripotency culture conditions. All clusters contained similar percentages of WT and ARID1A^−/−^ hESCs, except cluster 10. Particularly, over 90% cells in cluster 10 were from ARID1A^−/−^ hESCs (Fig. 2f). Cluster 10 had non-detectable level of ARID1A, but expressed neural stem cell markers ZIC1 and SOX1, and neuron markers MAP2 and FABP7, higher than any other clusters (Fig. 2g). In addition, neural-associated markers, such as PRTG, CDH6, and PTN, were highly expressed in cluster 10 (Additional file 1: Fig. S2a). Gene Ontology (GO) (Fig. 2h), process network, and signaling pathway (Additional file 1: Fig. S2b) analyses found that the upregulated genes (Additional file 2: Table S1) in cluster 10 (ARID1A^−/−^ vs. WT) were significantly enriched in neural development events, such as nervous system development, neurogenesis, and brain and head development. The feature plots showed that cells positively expressing neuroectoderm/neural stem cell markers (ZIC1, PAX6, SOX1) and neuron markers (MAP2, FABP7, PRTG, CDH6, and PTN) were highly enriched in cluster 10 and globally distributed in other clusters of ARID1A^−/−^ hESCs (red plots, Fig. 2i, Additional file 1: Fig. S2c). Loss-of-ARID1A resulted in decreased expression of OCT4 but did not affect the expression levels of other pluripotency markers (Additional file 1: Fig. S2d). Interestingly, endoderm (Additional file 1: Fig. S2e), mesoderm, and cardiac marker genes were repressed in ARID1A1/1/ hESCs (Additional file 1: Figs. S2f-h). Finally, flow cytometry, qRT-PCR, and immunostaining were performed to verify results from scRNA-seq. Increased percentages of PAX6^+^, MAP2^+^, and TUJ1^+^ (Fig. 2j, k, Additional file 1: Fig. S3a-c) cells were detected in ARID1A^−/−^ vs. WT hESCs by antibody staining followed with flow cytometry. Expression levels of neural-associated markers, such as PAX6, SOX1, MAP2, ZIC1, and FABP7, were significantly upregulated in ARID1A^−/−^ hESCs than those in WT (Fig. 2l, Additional file 1: Fig. S3d). PAX6^+^, SOX1^+^, MAP2^+^ (Fig. 2m), and TUJ1^+^ (Additional file 1: Fig. S3e) cells were detected by fluorescent immunostaining in ARID1A^−/−^ hESCs, but not in WT hESCs. Collectively, these results demonstrate that loss-of-ARID1A in hESCs increases transcription of neural genes and induces spontaneous neural differentiation (Fig. 2i, Additional file 1: Fig. S3f), indicating ARID1A plays an important role in controlling neurogenesis from hESCs.

### ARID1A oppositely controls cardiac and neural development from hESCs

**Fig. 3:**
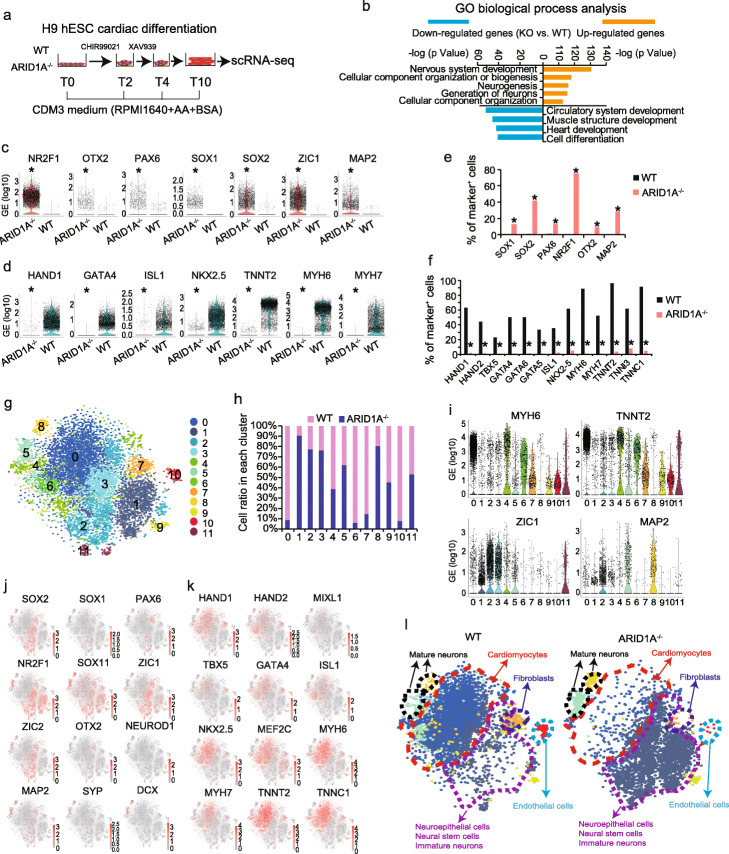
scRNA-seq reveals loss-of-ARID1A represses cardiac but promotes neural differentiation from hESCs. **a** A chemically defined cardiac differentiation protocol to induce cardiomyocyte differentiation from hESCs, followed with scRNAseq. **b** GO biological process analysis of upregulated and downregulated genes (KO vs. WT differentiated cells) by Metacore software. **c**, **d** Violin plots showing expression of neural-associated markers (**c**) and cardiac-associated markers (**d**) in WT and ARID1A^−/−^ derived cells. *p < 8.32E−100 (Wilcoxon’s test). **e**, **f** Comparison of percentages of WT and ARID1A^−/−^ differentiated cells which positively express neural-associated markers (**e**) and cardiac-associated markers (**f**). *p < 3.13E−163 (Fisher’s exact test) (**e**), *p < 1.4E−114 (Fisher’s exact test) (**f**). **g** Integrative analysis of scRNA-seq datasets of differentiated WT and ARID1A^−/−^ hESCs. Cell clusters were visualized with t-distributed stochastic neighbor embedding (t-SNE). **h** Integrative analysis showing the ratios of WT and ARID1A^−/−^ cells in each cluster of differentiated cells. **i** Violin plots showing expressions of cardiac- and neural-associated markers in all clusters. **j**, **k** Feature plots showing the expressions and distributions of cells positively expressing neural-associated markers (**j**) and cardiac-associated markers (**k**) in the integrated view. **l** Defined cell types in the separated WT (left) and ARID1A^−/−^ (right) cell clusters. WT (left) clusters contain most cardiomyocytes, and ARID1A^−/−^ (right) clusters contain most neural cells, indicating loss-of-ARID1A represses cardiac differentiation and promotes neural differentiation

We then asked whether loss-of-ARID1A could affect cardiac and neural differentiation from hESCs. A monolayer differentiation method [30] was utilized to induce cardiac differentiation from WT and ARID1A^−/−^ hESCs (Fig. 3a) for 10 days (T10), followed with scRNA-seq analyses to identify cellular and transcriptional heterogeneities. scRNA-seq revealed no ARID1A expression in ARID1A^−/−^ hESCs post-cardiac differentiation (Additional file 1: Fig. S4a). By conducting Gene Ontology (GO) (Fig. 3b) and process network (Additional file 1: Fig. S4b) analyses of all differentially expressed genes in ARID1A^−/−^ vs. WT cultures (Additional file 2: Table S1), we found that upregulated genes were significantly enriched for neural commitment events such as nervous system development, neurogenesis, and neural differentiation signaling pathway, whereas the downregulated genes were significantly enriched for cardiac commitment events, including circulatory system development, heart development, cardiac development, and cardiac myogenesis signaling pathways. For example, loss-of-ARID1A led to increased expression of essential neural marker genes (NR2F1, OTX2, PAX6, SOX1, SOX2, ZIC1, and MAP2) (Fig. 3c) and reduced expression of cardiac marker genes (HAND1, GATA4, ISL1, NKX2-5, TNNT2, MYH6, and MYH7) (Fig. 3d) at day 10 of differentiation. Next, we quantified the percentages of single cells which positively expressed neural and cardiac markers. Ratios of SOX1^+^, SOX2^+^, PAX6^+^, NR2F1^+^, OTX2^+^, and MAP2^+^ cells were higher in ARID1A^−/−^ cells than in WT cells (*p* < 3.13E−163, Fig. 3e). In contrast, ratios of cells expressing cardiac markers, such as HAND1/2, TBX5, GATA4/5/6, ISL1, NKX2-5, MYH6/7, TNNT2, TNNI3, and TNNC1, were significantly lower in differentiated ARID1A^−/−^ than in WT hESCs (*p* < 1.4E−114, Fig. 3f). scRNA-seq data from WT and ARID1A^−/−^ cells were integrated to generate 12 clusters, which were then interrogated with t-SNE analyses (Fig. 3g, Additional file 1: Fig. S4c) [28, 29]. Next, the ratios of ARID1A^−/−^ and WT cells in each cell cluster were analyzed (Fig. 3h). Approximately 90% of cells in clusters 0, 6, 7, and 10 and 60% of cells in cluster 4 were derived from WT hESCs, whereas about 80% of cells in clusters 1, 2, 3, and 8 and 60% of cells in cluster 5 were derived from ARID1A^−/−^ hESCs. Cells expressing CM-specific marker genes, such as MYH6, TNNT2 (Fig. 3i), NKX2-5, GATA4/6, TBX5, MEF2C, MYH7, TNNI3, and TNNC1 (Additional file 1: Fig. S4d), were enriched in clusters 0, 4, and 6. Neural stem cell/neuroepithelial cell marker genes, such as ZIC1 (Fig. 3i), NR2F1, NR6A1, PAX6, SOX2/11, and ZIC2 (Additional file 1: Fig. S4e), were highly expressed in cells of clusters 1, 2, and 3. Additionally, cells expressing neuron marker genes MAP2 (Fig. 3i), RBFOX3, SYP, and DCX (Additional file 1: Fig. S4e) were enriched in clusters 5 and 8. Average gene expression in each cell cluster (ARID1A^−/−^ vs. WT, Additional file 1: Figs. S4f-k) was consistent with the results of violin plots. We conducted GO biological process and signaling pathway analyses of upregulated genes in each cluster. Cardiac/neural development events and pathways were separately enriched into clusters (Additional file 1: Fig. S5). Feature plots from integrative analysis further distinguished distribution patterning of cells, which expressed signature neural and cardiac genes, across all clusters (Fig. 3j, k). Overall, scRNA-seq analysis found knockout-of-ARID1A predominantly enhanced differentiation of neural cells including neural stem cell, neuroepithelial cell, and mature neuron from hESCs, whereas differentiation of cardiac cells including cardiomyocyte, endothelial cell, and fibroblast was significantly suppressed (Fig. 3l). Given that cardiomyocyte, endothelial cell, and fibroblast originate from mesodermal cells, these results imply that loss-of-ARID1A could repress expression of genes controlling mesoderm formation.

**Fig. 4:**
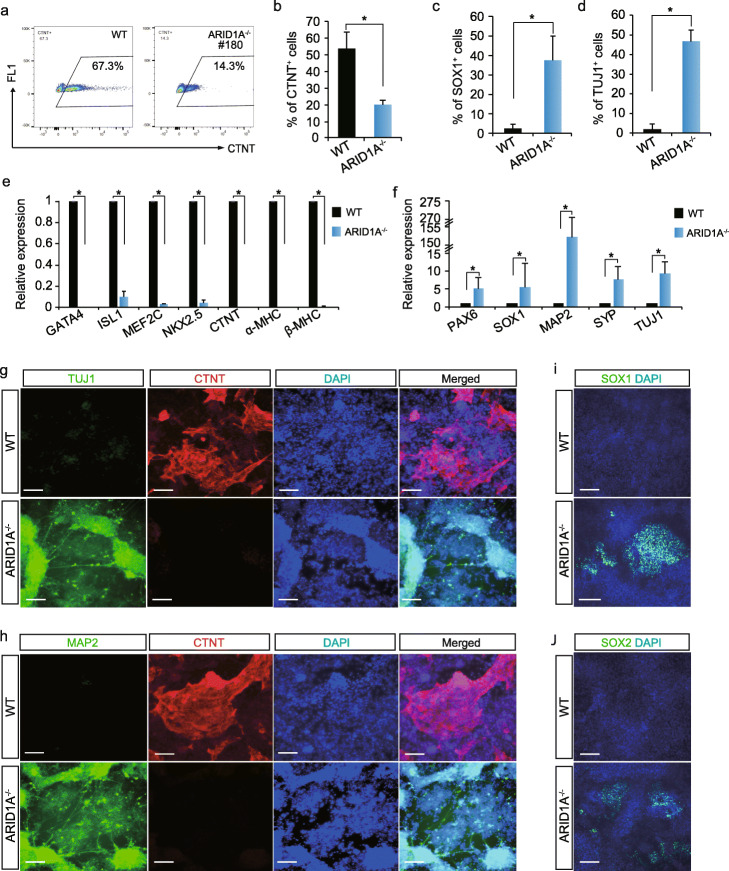
Characterization of WT and ARID1A^−/−^ hESC-derived cells. **a**, **b** Flow cytometry quantification of the ratios of CTNT^+^ CMs derived from WT and ARID1A^−/−^ cells after 10 days cardiac differentiation. All bars are shown as mean ± SD. *n* = 3, *p < 0.05 (an unpaired two-tailed *t* test). **c**, **d** Detecting ratios of SOX1^+^ (**c**) and TUJ1^+^ (**d**) cells in WT and ARID1A^−/−^ hESC-derived cells by immunostaining. All bars are shown as mean ± SD. *n* = 3, *p < 0.05 (an unpaired two-tailed *t* test). **e**, **f** Comparing the expression levels of cardiac markers (**e**) and neural markers (**f**) in WT and ARID1A^−/−^ hESC-derived cells by qRT-PCR. All bars are shown as mean ± SD. *n* = 3, *p < 0.05 (KO vs. WT for each gene, an unpaired two-tailed *t* test). **g**, **h** Immunostaining of neuron markers TUJ1 (**g**) and MAP2 (**h**) and cardiomyocyte marker CTNT (**g**, **h**) in WT and ARID1A^−/−^ hESC-derived cells after 10 days cardiac differentiation. Nuclei were labeled by DAPI. Scale bar, 100 μm. **i**, **j** Immunostaining of neural stem cell markers SOX1 (**i**) and SOX2 (**j**) in WT and ARID1A^−/−^ hESC-derived cells. Nuclei were labeled by DAPI. Scale bar, 100 μm

WT and ARID1A^−/−^ hESC-derived cells were collected after 10 days of cardiac differentiation for FACS, qRT-PCR, and immunostaining assessments (Fig. 3a). By performing FACS analysis, we found over 50% CTNT^+^ CMs from WT hESCs, whereas less than 20% CTNT^+^ CMs from ARID1A^−/−^ hESCs (Fig. 4a, b). Differential CM content in the cultures was also apparent by grossly monitoring contractile activity (Additional file 3: contractile activity videos of differentiated WT and ARID1A KO hESCs). However, over 30% of cells in cultures of ARID1A^−/−^ hESCs subjected to cardiac-directed differentiation were SOX1^+^ and TUJ1^+^as compared to less than 3% in cultures of WT hESCs (Fig. 4c, d). qRT-PCR analysis found loss-of-ARID1A significantly repressed cardiac (Fig. 4e) but prominently increased neural marker gene expression after differentiation (Fig. 4f). Immunomicroscopy detected a large number of CTNT^+^ CMs (Fig. 4g, h) and undetectable numbers of TUJ1^+^, MAP2^+^_,_ SOX1^+^, and SOX2^+^ neural cells (Fig. 4g–j) derived from WT hESCs. In contrast, cells derived from ARID1A^−/−^ hESCs gave opposite results (Fig. 4g–j). Next, a neural-specific differentiation protocol was performed to test whether loss-of-ARID1A can specifically affect neural commitment (Additional file 1: Fig. S6a). We found ARID1A^−/−^ hESCs gave rise to significantly increased percentages of PAX6^+^ and SOX1^+^ cells than WT hESCs by FACS analysis (Additional file 1: Figs. S6b-c) and immunostaining (Additional file 1: Figs. S6d-e). Expression levels of neural markers, such as PAX6, SOX1, and NEUROD1, increased after neural differentiation of ARID1A^−/−^ hESCs compared with WT hESCs (Additional file 1: Fig. S6f). Enhanced neural differentiation was observed in ARID1A^−/−^ hESCs under both neural and cardiac differentiation conditions, indicating a primary role of ARID1A in governing neural lineage specification from hESCs. To determine the relationship between the level of ARID1A expression and lineage commitment from hESCs, a single gRNA targeting the transcriptional start site of ARID1A was used in combination with CRISPR/Cas9 (Additional file 1: Fig. S6g) to knock down ARID1A expression to 60% of that in WT hESCs (Additional file 1: Fig. S6h). After 10 days of cardiac differentiation, we found ARID1A^knockdown^ hESCs gave rise to half of CTNT^+^ CMs derived from WT hESCs (Additional file 1: Fig. S6i). Additional experiments using shRNA-mediated knockdown of ARID1A (Additional file 1: Fig. S6j) confirmed that downregulation of ARID1A suppressed CM differentiation (Additional file 1: Fig. S6k). However, no spontaneously differentiated neural cells were found in ARID1A^knockdown^ hESCs maintained in pluripotent culture conditions (Additional file 1: Fig. S6l). Together, these results demonstrate that cardiac differentiation efficiency from hESCs is dependent on the dose of ARID1A, whereas spontaneous neural differentiation is blocked by the presence of ARID1A, implying different mechanisms by which ARID1A controls human cardiogenesis and neurogenesis.

### ATAC-seq reveals global alterations of chromatin remodeling in ARID1A^−/−^ hESCs

**Fig. 5:**
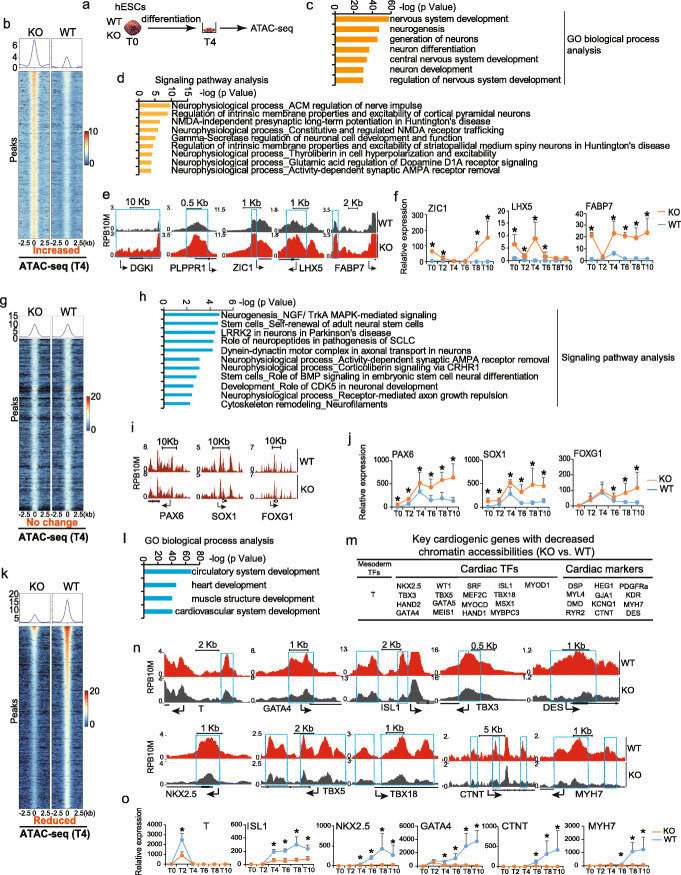
Loss-of-ARID1A leads to changes of chromatin accessibility. **a** Assay for Transposase-Accessible Chromatin with high-throughput sequencing (ATAC-seq) was performed after 4 days differentiation of WT and ARID1A^−/−^ hESCs. **b** Heatmap and pileup of ATAC-seq signal for genes with increased chromatin accessibility (KO vs. WT). **c**, **d** GO biological process (**c**) and signaling pathway enrichment (**d**) analysis of genes with increased chromatin accessibility (KO vs. WT). The analysis of GO biological process and signaling pathways was performed by Metacore software. **e** Genome views of neurogenic genes with increased chromatin accessibility (KO vs. WT) close to transcription start site (TSS). Arrows showed the transcriptional direction. **f** qRT-PCR detection of neurogenic gene expression profiles during cardiac differentiation of hESCs. **g** Heatmap and pileup of ATAC-seq signals for genes without significant changes on chromatin accessibility (KO vs. WT). **h** Signaling pathways enriched in genes with no-changed chromatin accessibility (KO vs. WT). The analysis of signaling pathways was performed by Metacore software. **i** Genome views of neurogenic genes without significant changes on chromatin accessibility (KO vs. WT). **j** qRT-PCR detection of neurogenic gene expression profiles during cardiac differentiation of hESCs. **k** Heatmap and pileup of ATAC-seq signals for genes with reduced chromatin accessibilities (KO vs. WT). **l** GO biological process analysis of genes with reduced chromatin accessibilities (KO vs. WT). The analysis of GO biological process was performed by Metacore software. **m** List of essential cardiogenic and cardiac functional genes showing decreased chromatin accessibility in KO cells (KO vs. WT). **n** Genome views of some important cardiogenic genes with decreased chromatin accessibility in KO cells compared to WT. Arrows showed the transcriptional direction. **o** qRT-PCR detection of cardiogenic gene expression profiles during cardiac differentiation of hESCs. All bars in RT-qPCR data are shown as mean ± SD. *n* = 3, *p < 0.05 (KO vs. WT, an unpaired two-tailed *t* test with Welch’s correction)

Given that SWI/SNF complex remodels chromatin to facilitate transcription of target genes, we performed ATAC-seq to investigate the impact of ARID1A on chromatin accessibility during cardiogenesis. hESCs at the day 4 (T4) of cardiac differentiation were collected for ATAC-seq (Fig. 5a). Global changes of chromatin accessibility, including increased (Fig. 5b), no change (Fig. 5g), and reduced (Fig. 5k), in ARID1A^−/−^ vs. WT hESCs were detected (Additional file 4: Table S2). GO analysis identified genes associated with differential chromatin accessibility changes have distinct biological functions. Genes with increased (Fig. 5b) or no change (Fig. 5g) of chromatin accessibility (ARID1A^−/−^ vs. WT) were implicated in neural commitment events such as nervous system development and neurogenesis (Fig. 5c, d, h, Additional file 1: Fig. S7a). As shown by ATAC-seq (Fig. 5e) and qRT-PCR (Fig. 5f) analyses, loss-of-ARID1A increased both chromatin accessibility and expression levels of neurogenic genes, such as ZIC1, LHX5, and FABP7. However, chromatin accessibility on other neurogenic genes, such as PAX6, SOX1, and FOXG1 (Fig. 5i), exhibited no obvious changes (ARID1A^−/−^ vs. WT), albeit their significantly increased expression levels (Fig. 5j). These results suggest that transcriptional activities, rather than chromatin accessibility, of critical neurogenic genes were dependent on ARID1A. Notably, genes with reduced chromatin accessibility (ARID1A^−/−^ vs. WT) (Fig. 5k) were mainly enriched in cardiac commitment events, such as circulatory, heart, and cardiovascular system development (Fig. 5l, Additional file 1: Fig. S7b). These genes included early mesoderm transcriptional factors T [31–37]; cardiac transcription factors GATA4, ISL1, TBX3, DES, NKX2.5, TBX5, and TBX18; and sarcomeric genes CTNT and MYH7 (Fig. 5m, n). Additionally, during cardiac differentiation, expression levels of these cardiogenic genes were all significantly decreased in ARID1A^−/−^ cells compared with WT cells (Fig. 5o). Loss-of-ARID1A did not affect the expression of endoderm marker genes, such as SOX17 and FOXA2 (Additional file 1: Fig. S7c). Altogether, these results indicate that loss-of-ARID1A globally reduces chromatin accessibility on essential cardiogenic genes, but not on neurogenic genes, whereas transcription of essential neurogenic genes is dependent on ARID1A.

### ChIP-seq reveals genome-wide ARID1A occupancy on promoters of essential cardiogenic and neurogenic genes

**Fig. 6:**
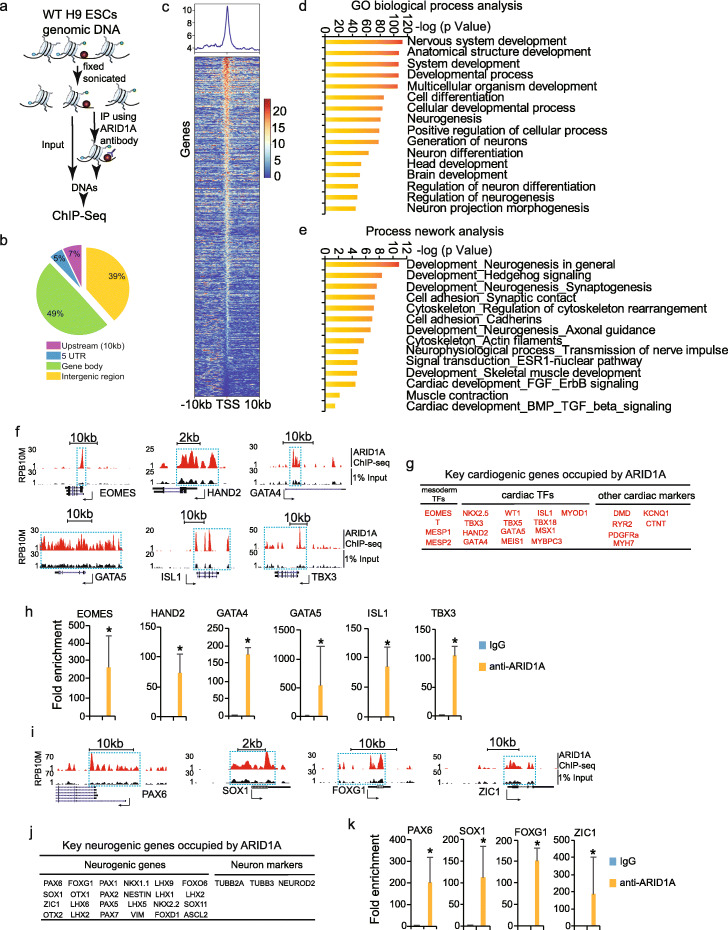
ChIP-seq reveals genome-wide ARID1A occupancies on promoters of cardiogenic and neurogenic genes. **a** Chromatin immunoprecipitation (ChIP) assays with sequencing (ChIP-Seq) was performed in WT H9 hESCs. **b** Distribution of ARID1A ChIP-seq-enriched peaks across human genome. **c** Relative enrichment levels of ARID1A on transcription start site (TSS) of coding genes in hESCs. Heatmaps were ranked by ARID1A enrichment. **d** GO biological process analysis of genes enriched by ARID1A ChIP-seq. The analysis of GO biological process was performed by Metacore software. **e** Process network analysis of genes enriched by ARID1A ChIP-seq. The analysis of process networks was performed by Metacore software. **f** Example genome views of ARID1A occupancies on mesodermal TF EOMES, cardiac TFs HAND2, GATA4, GATA5, ISL1, and TBX3. **g** List of essential cardiogenic and cardiac functional genes occupied by ARID1A. **h** ChIP-qPCR validation of cardiogenic gene promoters occupied by ARID1A. **i** Example genome views of ARID1A occupancies on neurogenic genes. **j** List of essential neurogenic and neural marker genes occupied by ARID1A. **k** ChIP-qPCR validation of neurogenic gene promoters occupied by ARID1A. All bars in ChIP-QPCR data are shown as mean ± SD. *n* = 3, *p < 0.05 (anti-ARID1A vs. IgG, an unpaired two-tailed *t* test)

Given the DNA binding capacity of ARID1A [15], chromatin immunoprecipitation assay with sequencing (ChIP-seq) was next carried out to define genome-wide ARID1A occupancy in hESCs (Fig. 6a). Approximately 61% of ARID1A binding sites located on regions within 10 kb from transcriptional start sites (TSS), 5′UTR, and gene bodies (Fig. 6b). Interestingly, GO analyses revealed that genes with ARID1A occupancies close to TSS (Fig. 6c, Additional file 5: Table S3) were enriched in both neural and cardiac commitment, such as nervous system development, neurogenesis (Fig. 6d), and cardiac development/muscle contraction (Fig. 6e). For example, the gene encoding a transcriptional factor for early mesodermal commitment, EOMES, and genes encoding key cardiac transcriptional factors, HAND2, GATA4, GATA5, ISL1, and TBX3, were all occupied by ARID1A on their promoter regions (Fig. 6f, g), which were confirmed by ChIP-qPCR (Fig. 6h). Both ChIP-seq and ChIP-qPCR also identified ARID1A-occupied neurogenic genes, such as PAX6, SOX1, FOXG1, and ZIC1 (Fig. 6i–k). Importantly, by comparing the ATAC-seq (Fig. 5m) and ARID1A ChIP-seq data (Fig. 6g), we found all ARID1A-occupied cardiogenic genes exhibited decreased chromatin accessibility during cardiac commitment from ARID1A^−/−^ vs. WT hESCs, indicating that chromatin accessibility of cardiogenic genes was dependent on ARID1A. Although ARID1A occupied promoters of many neurogenic genes (Fig. 6j), most of them, except several genes including ZIC1, LHX5, and FABP7 (Fig. 5e), did not exhibit significant alternations of chromatin accessibility under ARID1A deficiency. This suggests that ARID1A globally occupies neurogenic gene promoters to suppress transcription via a non-chromatin-remodeling mechanism. It also implies the existence of ARID1A-recruited co-factor(s) to repress neurogenic gene transcription.

### ARID1A associates with different transcriptional factors

**Fig. 7:**
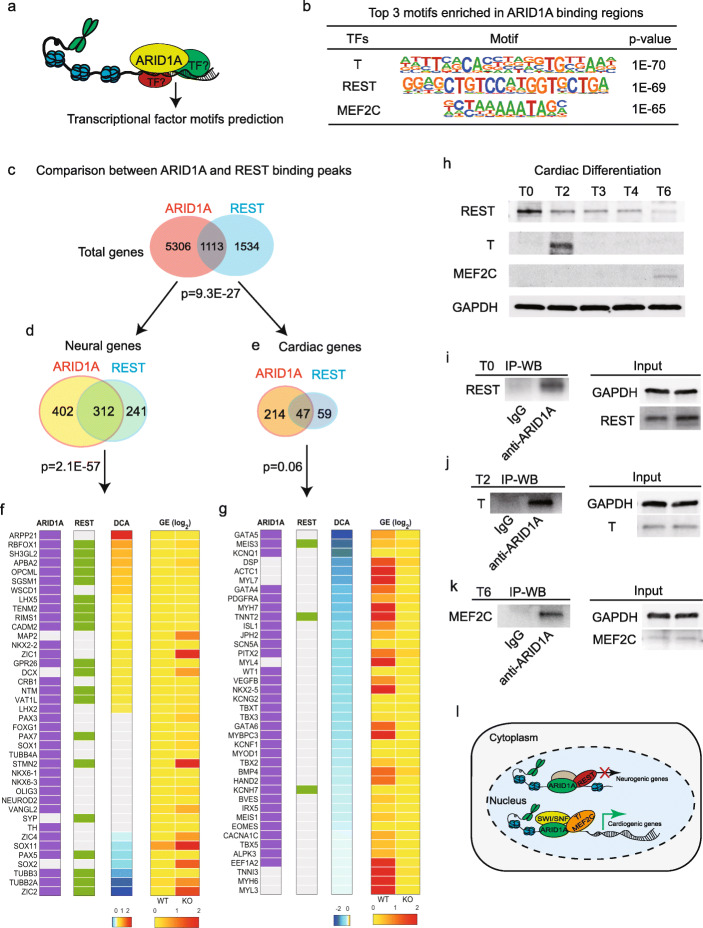
ARID1A interacts with co-factors. **a** Prediction of co-factors interacting with ARID1A according to transcriptional factor binding motifs in ARID1A ChIP-seq peaks. **b** Top three transcription factor motifs surrounding ARID1A cis-regulatory binding elements. **c** Comparison between ARID1A-and REST-occupied gene lists from ChIP-seq datasets. Hypergeometric model was used to calculate the significance of overlap between ARID1A and REST target genes, and *p* value of enrichment of genes in both ARID1A and REST targets. **d**, **e** Comparison between ARID1A- and REST-occupied neural (**d**) and cardiac genes (**e**) from ChIP-seq datasets, indicating that REST and ARID1A prefer to co-occupy neural genes than cardiac genes. **f** Integrative analysis of ARID1A ChIP-seq (1st column), REST ChIP-seq (2nd column), differential chromatin accessibility (DCA) by ATAC-seq (KO vs. WT) (3rd column), and average gene expression levels (GE, log2 (fold change)) from scRNA-seq (KO vs. WT, 4th and 5th columns). Purple boxes (1st column) show the representative ARID1A-occupied genes from **d**, while green boxes (2nd column) show REST occupancies on those genes. **g** Same scheme design as **f** to show ARID1A and REST occupancies on representative cardiogenic genes are shown. **h** Western blotting to detect temporal protein expressions of REST, T, and MEF2C during cardiac differentiation of hESCs. Protein samples were collected on days 0 (T0), 2 (T2), 3 (T3), 4 (T4), and 6 (T6). **i**–**k** Co-IP validation of the interaction between ARID1A and REST in undifferentiated hESCs (T0) (**i**), the interaction between ARID1A and T in hESCs at day 2 of differentiation (T2) (**j**), and the interaction between ARID1A and MEF2C in hESCs at day 6 of differentiation (T6) (**k**). **l** Schematic summary of ARID1A’s roles in human cardiac and neural commitment from hESCs

In order to define co-factors that interact with ARID1A, we predicted potential co-TFs based on their motif sequences within the ARID1A-occupied genomic regions from ARID1A ChIP-seq (Fig. 7a). TBXT (T), repressor element-1 silencing transcription/neuron-restrictive silencer factor (REST/NRSF), and MEF2C were the top three predicted co-TFs (Fig. 7b). REST/NRSF was known to specifically repress neural gene transcription [38]. We then reanalyzed the published REST/NRSF ChIP-seq data (ENCODE: ENCSR663WAR) generated in H1 hESCs. A notable overlap was observed between REST- and ARID1A-occupied genes (Fig. 7c). There were total 1113 genes co-occupied by ARID1A and REST (*p* = 9.3E−27). 28.0% (312 out of 1113) of the co-occupied genes were neural (*p* = 2.1E−57) (Fig. 7d), and 4.2% (47 out of 1113) were cardiac (*p* = 0.06) (Fig. 7e), indicating a strong bias that ARID1A and REST tend to co-occupy more neural genes than cardiac genes. The lists of neural and cardiac genes were from an unbiased database, the Human Protein Atlas database (https://www.proteinatlas.org/). Next, integrated data analyses were performed on 40 essential neurogenic (Fig. 7f) and cardiogenic genes (Fig. 7g) showing descending differential chromatin accessibility (DCA). The purple (1st column) and green (2nd column) bars represented whether the gene was occupied by ARID1A and REST, respectively. The 3rd column in Fig. 7f, g indicated signal differences of DCA during cardiac commitment. The two columns on the right (4th and 5th column) showed relative gene expressions in WT and ARID1A^−/−^ hESC-derived cells post-cardiac differentiation. In agreement with the results shown in Figs. 7d, e, REST and ARID1A co-occupied more neurogenic genes (Fig. 7f) than cardiogenic genes (Fig. 7g). REST and ARID1A co-occupied neural genes which showed various changes of chromatin accessibility, including increased, decreased, or non-changed accessibility (Fig. 7f, DCA). However, expression levels of all these neurogenic genes increased in the middle of (Fig. 5f, j) and after differentiation of ARID1A^−/−^ cells when compared with WT cells (Fig. 7f, 4th and 5th column). These results suggest that ARID1A-REST/NRSF interaction represses transcription of co-occupied neurogenic genes. In contrast, REST barely co-occupied with ARID1A on promoter regions of essential cardiogenic genes in Fig. 7g.

Interactions of ARID1A with REST, T, and MEF2C were confirmed using co-immunoprecipitation (Co-IP) assay. Western blotting was performed to detect temporal protein levels of REST, T, and MEF2C during cardiac differentiation of WT hESCs (Fig. 7h). REST was expressed at all time points, with gradual decrease in levels as differentiation progressed. T showed peak expression at day 2 (T2) of mesoderm formation. MEF2C, a key cardiac TF, was expressed from day 6 (T6). REST was present in ARID1A immune complex generated from hESCs (Fig. 7i). Moreover, anti-ARID1A antibody could pull down T at day 2 (Fig. 7j) and MEF2C at day 6 (Fig. 7k) of cardiac differentiation, and vice versa (Additional file 1: Figs. S7d-e), suggesting that ARID1A could subsequently interact with T and MEF2C to regulate chromatin accessibility during human heart development. Taken together, our results reveal the molecular mechanisms by which ARID1A regulates early human cardiac and neural development from hESCs (Fig. 7l).

## Discussion

In this study, we found ARID1A promoted human cardiac commitment via increasing chromatin accessibility of essential cardiogenic genes and suppressed neural commitment via repressing transcription of neurogenic genes. Thus, ARID1A is able to coordinate human cardiogenesis and neurogenesis from pluripotent stem cells via distinct molecular mechanisms.

The classical function of SWI/SNF complex is to remodel chromatin state and DNA accessibility to transcription factors and hence is critical for transcriptional regulation and gene expression. Arid1a (Baf250a) has been previously reported to play important role in mouse heart formation. Loss-of-Arid1a resulted in embryonic lethality with lack of primitive streak or mesoderm formation [15, 17]. Arid1a ablation in mouse second heart field (SHF) leads to trabeculation defects in the right ventricle [39]. Although previous in vivo studies revealed a critical role of Arid1a in mouse heart morphogenesis, how ARID1A drives early stage human cardiogenesis from pluripotent stem cells remains elusive. Our data demonstrate that ARID1A promotes human cardiac development from hESCs by globally increasing chromatin accessibility on essential cardiogenic genes, which include key mesoderm formation genes, cardiac specific transcriptional factors, and sarcomeric genes. During cardiogenesis, temporal interaction of ARID1A was observed with the genes encoding T (a key regulator of mesoderm formation) and MEF2C (a key cardiac cardiomyogenic transcriptional factor). These interactions could very likely facilitate the sequential deposition of SWI/SNF complex on the genes, thereby favoring cardiomyogenic differentiation. Although additional studies are warranted, our data clearly indicate that early human cardiac fate commitment from hESCs is largely dependent on the chromatin-remodeling role of SWI/SNF complex.

SWI/SNF complex was also found to regulate neurodevelopment in mouse. Mice with loss of Brg [40], Baf47 (also known as Smarcb1) [41], or Baf155 (also known as Smarcc1) [42] die at the pre- or peri-implantation stage. Conditional deletion of Brg1 (an ATPase of SWI/SNF complex) in the developing mouse nervous system results in the formation of a smaller brain that lacks a cerebellum [43]. These neurodevelopmental defects could be due to failure of neural progenitor self-renewal and/or differentiation [43, 44]. However, in contrast to the role of Brg1 in mouse neural development, our data show that loss-of-ARID1A prominently enhanced neurogenesis from hESCs. Knockout-of-ARID1A in hESCs increased neural differentiation under both targeted cardiac and neural differentiation conditions. Since the cardiac differentiation protocol was specifically modified to support mesoderm and cardiomyocyte differentiation, the robust generation of neural cells from ARID1A^−/−^ hESCs under those conditions strongly suggests a master regulatory role of ARID1A in governing neurogenesis. The increased neural differentiation of ARID1A^−/−^ hESCs and underdeveloped brain in Brg1 KO mice indicate that ARID1A might have SWI/SNF-independent functions in neurogenesis. This was proved by our finding that ARID1A did not uniformly affect the chromatin accessibility on neurogenic genes, but rather globally controlled the transcriptional activity of neurogenic genes. We found loss-of-ARID1A induced sporadic neural differentiation together with increased expression of multiple neurogenic genes in hESCs. Given the heterogeneity of hESCs, this observation suggests that the chromatin accessibility of neurogenic genes in subpopulations of undifferentiated hESCs is very likely in a relatively open configuration. Thus, loss-of-ARID1A (and consequently the ability to differentially remodel chromatin structure) would favor default transcriptional activation of those genes. In fact, multiple previous studies reported that neural-associated genes in hESCs are under a bivalent condition with enhanced chromatin and transcriptional plasticity, and tend to be transcribed [45–47].

It has previously been shown in mice that disruption of Arid1a-DNA interaction by a single nucleotide variant (Arid1aV^1068G/V1068G^) [15] resulted in neural tube defects including open head folds, neural tube closure defects, and heart defects such as trabeculation defects, hypoplastic myocardial walls, and ventricular septal defects. Although it was not defined whether the neural tube closure defects in Arid1aV^1068G/V1068G^ mouse were due to increased neurogenesis, it was previously reported that an increased number of neural stem cells will lead to defective neural tube closure [48–50]. Our ATAC-seq and ChIP-seq in hESCs revealed that loss-of-ARID1A globally reduced chromatin accessibility on promoters of cardiogenic genes and activated transcription of neurogenic genes. Taken together, the results from Arid1aV^1068G/V1068G^ mouse model and the ARID1A^−/−^ hESCs data presented here demonstrate that ARID1A plays a conserved and critical role in controlling both cardiogenesis and neurogenesis.

Our data show that ARID1A interacts with REST/NRSF. During embryogenesis, REST/NRSF is widely expressed and plays a critical role in neuronal development. In pluripotent stem cells and neural progenitors, REST represses a large pool of neuron-specific genes essential for synaptic plasticity and remodeling, including synaptic vesicle proteins, neuroreceptors, and ion channels [51–53]. The mechanism by which ARID1A-REST recognizes target neurogenic genes remains elusive. A previous report in HEK293 cells found that BRG1 enhanced REST-mediated repression of some target genes by increasing the binding of REST with local chromatin [54]. Whether this was mediated via REST-ARID1A interaction was not clear. Additionally, Pax6 (a master neuroregulatory gene) interacts with neural progenitor BAF (npBAF) complex to regulate neurogenesis [55]. Therefore, it is likely that Pax6 (or other TFs) could guide ARID1A-REST to target neurogenic genes during differentiation of hESCs. The observation that ChIP-seq revealed ARID1A occupancy on essential cardiogenic and neurogenic genes in undifferentiated hESCs suggests that cardiogenesis and neurogenesis may be pre-determined by epigenetic machineries at the pluripotent stem cell stage. This also suggests that ARID1A-SWI/SNF could associate with factor(s), other than REST, to repress spontaneous cardiac differentiation in hESCs.

## Conclusion

This study uncovers the essential and opposite roles of ARID1A in governing cardiogenesis and neurogenesis from human pluripotent stem cells. Our findings reveal the distinct mechanisms by which ARID1A globally enhances chromatin remodeling on cardiogenic genes and suppresses transcription of neurogenic genes.

## Methods

### Human embryonic stem cell culture, cardiac differentiation, and neural differentiation

Human embryonic stem cell (hESC) line H9 was cultured on Matrigel (BD Biosciences)-coated plates in mTesR medium [56, 57]. For cardiac differentiation, a monolayer differentiation method was used as described and reported [30]. Briefly, hESCs were induced with chemically defined medium (CDM3, containing RPMI/1640, AA, and BSA) as the basal medium [30] containing 6 μM CHIR99021 (Tocris Bioscience) from day 0 to day 2 and 5 μM XAV-939 (Tocris Bioscience) from day 2 to day 4, then maintained in CDM3 basal medium without any chemicals for total 10–13 days. Beating cardiomyocytes were observed after differentiation for 8 to13 days. For neural differentiation, H9 hESCs were dissociated and replated into 6-well plate (coated with metrigel) and induced in N2B27 medium (25 mL DMEM/F12 medium, 1× N2, 25 mL neurobasal medium, 1× B27, 1× Glutamax, 1× NEAA, and 1× penicillin-streptomycin). After 4 days differentiation, cells were collected for experiments.

### Vector cloning

Specific shRNA1 and shRNA2 targeting human ARID1A were cloned into the pLKO.1-TRC-puro vector (Addgene), separately. shRNA scramble, as a negative control without targeting any human genes, was also cloned into pLKO.1-TRC-puro vector.

### CRISPR/Cas-9 mediated DNA manipulation

All gRNAs were designed based on CRISPR design platform from MIT (http://crispr.mit.edu/). Full length of human ARID1A gene was knocked out by dual gRNAs in hESC H9. For each gRNA oligo, self-complementary oligos were purchased from Invitrogen. Both of gRNAs, targeting 5′ sequence and 3′ sequence of ARID1A DNA, were cloned into the pENTR-spCAS9-T2A-EGFP vector (from Dr. Yi Sheng), separately. Single gRNA, targeting and disrupting ARID1A TSS, was cloned into the lentiCRISPRv2-puro vector (Addgene) [58]. pENTR-spCAS9-T2A-EGFP-gRNA vectors were transfected into H9 hESCs using X-treme GENE 9 DNA Transfection Reagent (Roche). After 24 h transfection, GFP positive cells were sorted by FACSAria II cell sorter (BD Biosciences) and replated to grow single clones in mTesR medium with ROCK inhibitor Y-27632 (Tocris Bioscience). Single H9 clones were picked out and replated into 48-well plate for further expansion. Genomic DNAs of single H9 clones were extracted by the DNeasy Blood & Tissue kit (Qiagen) kit. Null ARID1A hESC clones were verified by PCR using different primer sets. For ARID1A TSS disruption, H9 cells were infected with lentiCRISPRv2-puro-ARID1A gRNA virus and puromycin was added to select drug-resistant clones. LentiCRISPRv2-puro-ARID1A gRNA vector-mediated genomic DNA editing on human ARID1A promoter was detected by Surveyor® Mutation Detection Kit for Standard Gel Electrophoresis (Integrated DNA Technologies, Inc.).

### Surveyor assay

Genomic DNA editing was detected using Surveyor® Mutation Detection Kit for Standard Gel Electrophoresis (Integrated DNA Technologies) according to manufacturer’s instructions. Briefly, genomic DNA was extracted by DNeasy Blood & Tissue Kit (Qiagen). PCR fragments were purified by GeneJET PCR Purification Kit (K0701). After that, PCR fragments were annealing in 1× prime star buffer (Takara) and Surveyor enzyme was added to digest the annealed DNA fragments at 42 °C for 1 h. Gel Electrophoresis on agarose gel was used to detect the digestion.

### Lentivirus package and transduction

The lentiviral vector lentiCRISPRv2-puro was transfected into the HEK293T cells (ATCC) along with packaging plasmids including psPAX2 and pMD2.G (from Dr. Guang Hu in NIH) using the X-treme GENE 9 transfection reagent (Roche). Viral supernatant was collected, and cellular debris was removed by syringe filtering (0.45 μm pore size; Millipore). Human H9 cells cultured in mTesR medium were incubated with virus media for 4 h, followed with fresh mTesR medium culture for overnight. The same infection was repeated after 24 h. Puromycin was added to select puromycin-resistant H9 clones after virus infection for 48 h.

### PCR screening of ARID1A^−/−^ clones

hESC clones were picked and expanded in mTesR medium. Genomic DNAs from each hESCs clone were purified by DNeasy Blood & Tissue Kits (Qiagen). PCRs with different primer sets were conducted by using DreamTaq Green PCR Master Mix (Thermo Scientific) or PrimeSTAR DNA Polymerase kit (Takara). PCR products were run on agarose gel to visualize the fragment size. For all primers, please see Additional file 6: Table S4.

### RNA extraction and RT-quantitative PCR

Total RNAs were extracted by miRNeasy mini kit (Qiagen) or RNeasy Mini Kit (Qiagen). High-Capacity RNA-to-cDNA™ Kit (Applied Biosystems) was used for cDNA synthesis. RT-quantitative PCR (RT-qPCR) was performed on a 7900HT Fast Real-Time PCR System (Applied Biosystems) with Fast SYBR Green Master Mix (Applied Biosystems). The RT-qPCR results were normalized to internal control GAPDH or beta-ACTIN using the 2^−ΔΔCt^ method as previously described [59]. RT-qPCR data were presented as mean ± SD from at least three independent experiments.

### Western blotting

Cells were lysed, and proteins were purified by Complete™ Lysis-M EDTA-free kit (Roche, 04719964001). Protein samples were run on Mini-PROTEAN® TGX™ Precast Gels (Bio-Rad) and transferred to PVDF membranes by a wet transfer apparatus (Bio-Rad). After blotting with 5% non-fat milk for 30 min at room temperature, the membranes were incubated in TBXT buffer containing 5% non-fat milk and primary antibodies at 4 °C overnight. On the next day, membranes were incubated in TBXT buffer containing 5% non-fat milk and horseradish peroxidase (HRP)-conjugated anti-rabbit or anti-mouse IgG (Cell Signaling Tech.) and detected by using ECL Western Blotting Substrate (Pierce).

### Co-immunoprecipitation (Co-IP)

Cell proteins were extracted by Pierce™ Classic Magnetic IP/Co-IP Kit (Thermo Scientific, 88804) or Complete™ Lysis-M EDTA-free kit (Roche, 04719964001) according to the manuals. Protein co-immunoprecipitation (Co-IP) was performed by using Pierce™ Classic Magnetic IP/Co-IP Kit as well. Protein samples were run on Mini-PROTEAN TGX Precast Gels (Bio-Rad) and probed with appropriate antibodies by Western blotting.

### Chromatin immunoprecipitation-qPCR (ChIP-qPCR)

H9 hESCs were cultured in P10 plate in mTesRm medium. ChIP was carried out in undifferentiated hESCs according to manuals of truChIP™ Chromatin Shearing Kit (Covaris, PN 520154) and EZ-Magna ChIP™ A/G Chromatin Immunoprecipitation Kit (Millipore, 17-10086). Briefly, hESCs were fixed with methanol-free formaldehyde provided by truChIP™ Chromatin Shearing Kit (Covaris, PN 520154). Sonication of cell lysis was done by a ME220 Focused-ultrasonicator using truChIP Chromatin Shearing Tissue Kit (Covaris). Chromatin immunoprecipitation (ChIP) was performed by EZ Magna ChIP™ A/G Chromatin Immunoprecipitation Kit (Millipore). Human ARID1A antibody (Millipore: (04-080) Anti-BAF250a/ARID1a Antibody, clone PSG3) was used to pull down genomic DNAs. Normal mouse/rabbit IgG or RNA Polymerase II antibodies (provided by Millipore ChIP kit) were used as negative or positive control, respectively. ChIP-qPCR signals were calculated as fold enrichment of 1% input or non-specific antibody (isotype IgG antibodies) signals with at less three technical triplicates. Each specific antibody ChIP sample was normalized to its isotype IgG antibody-ChIP signals obtained in the same sample. Standard deviations (SD) were calculated from technical triplicates and represented as error bars.

### ChIP-sequencing (ChIP-seq)

H9 hESCs were cultured in P10 plate in mTesRm medium. ChIP was carried out in undifferentiated hESCs according to the manuals of truChIP™ Chromatin Shearing Kit (Covaris, PN 520154) and EZ-Magna ChIP™ A/G Chromatin Immunoprecipitation Kit (Millipore, 17-10086). Briefly, hESCs were fixed with methanol-free formaldehyde provided by truChIP™ Chromatin Shearing Kit (Covaris, PN 520154). Chromatin of cell lysis was sheared using truChIP™ Chromatin Shearing Kit according its manual by ME220 Focused-ultrasonicator (Covaris). The sheared chromatin was then incubated with anti-ARID1A antibody and purified by using EZ-Magna ChIP™ A/G Chromatin Immunoprecipitation Kit (Millipore, 17-10086). Chromatin DNA quality was assessed by 2100 Bioanalyzer (Agilent Technologies) and sequenced in the Center for Medical Genomics at Indiana University School of Medicine.

### Single-cell 3′ RNA sequencing (scRNA-seq)

H9 hESCs and differentiated hESCs were dissociated by Corning™ 0.25% Trypsin (Corning™ 25053CI) to single cells. Single-cell 3′ RNA-seq experiments were conducted using the Chromium single cell system (10X Genomics, Inc) and Illumina sequencers at the Center of Molecular Genetics (CMG) of Indiana University School of Medicine. Cell suspension was first inspected on the Countess II FL (Thermo Fisher Scientific) and under microscope for cell number, cell viability, and cell size. Depending on the quality of the initial cell suspension, the single-cell preparation included centrifugation, resuspension, and filtration to remove cell debris, dead cells, and cell aggregates. Single-cell capture and library preparation were carried out according to the Chromium Single Cell 3′ Reagent kits V2 User Guide (10X Genomics PN-120267, PN-1000009, PN-120262). Appropriate numbers of cells were loaded on a multiple-channel micro-fluidics chip of the Chromium Single Cell Instrument (10X Genomics) with a targeted cell recovery of 10,000. Single-cell gel beads in emulsion containing barcoded oligonucleotides and reverse transcriptase reagents were generated with the v2 single cell reagent kit (10X Genomics). Following cell capture and cell lysis, cDNA was synthesized and amplified. Illumina sequencing libraries were then prepared with the amplified cDNA. The resulting libraries were assessed with an Agilent TapeStation or Bioanalyzer 2100. The final libraries of the undifferentiated samples were sequenced using a custom program on Illumina NextSeq 500/550, and the libraries of the differentiated samples were sequenced on Illumina NovaSeq 6000. Twenty six base pairs of cell barcode and UMI sequences, and 91 or 98 bp RNA reads were generated with Illumina NextSeq500/550 or NovaSeq 6000.

### Analysis of scRNA-seq data

CellRanger 2.1.0 (http://support.10xgenomics.com/) was utilized to process the raw sequence data generated. Briefly, CellRanger used bcl2fastq (https://support.illumina.com/) to demultiplex raw base sequence calls generated from the sequencer into sample-specific FASTQ files. The FASTQ files were then aligned to the human reference genome GRCh38 with RNA-seq aligner STAR. The aligned reads were traced back to individual cells, and the gene expression level of individual genes was quantified based on the number of UMIs (unique molecular indices) detected in each cell. The filtered gene-cell barcode matrices generated with CellRanger were used for further analysis with the R package Seurat (version 2.3.1) [28] with Rstudio version 1.1.453 and R version 3.5.1. Quality control (QC) of the data was implemented as the first step in our analysis. We first filtered out genes that were detected in less than five cells and cells with less than 200 genes. To further exclude low-quality cells in downstream analysis, we used the function isOutlier from R package scater [29] together with visual inspection of the distributions of number of genes, UMIs, and mitochondrial gene content. Cells with extremely high or low number of detected genes/UMIs were excluded. In addition, cells with high percentage of mitochondrial reads were also filtered out. After removing likely multiplets and low-quality cells, the gene expression levels for each cell were normalized with the NormalizeData function in Seurat. To reduce variations sourced from different numbers of UMIs and mitochondrial gene expression, we used the ScaleData function to linearly regress out these variations. Highly variable genes were identified (x.low.cutoff = 0.0125, x.high.cutoff = 4, y.cutoff = 0.5).

To integrate the single-cell data from undifferentiated or differentiated WT and ARID1A^−/−^ samples, we applied the canonical correlation analysis (CCA) in Seurat. We chose the top 1500 variable genes from each sample to calculate the correlation components (CCs) and used the function MetageneBicorPlot to determine the optimal number of CCs. We retained the cells whose expression profiles could be explained with at least 50% by the CCs using CalcVarExpRatio and SubsetData. The CCA subspaces were then aligned with AlighSubspace using the number of CCs determined. We employed FindClusters for shared nearest neighbor (SNN) graph-based clustering. The clusters were visualized with t-distributed stochastic neighbor embedding (t-SNE) by running dimensionality reduction with RunTSNE and TSNEPlot. The FindConservedMarkers function was subsequently used to identify canonical cell type marker genes that are conserved across WT and knockout cells. To compare average gene expression within the same cluster between WT and knockout cells, we applied AverageExpression. R packages ggplot2 (ISBN 978-3-319-24,277-4) and ggrepel (https://github.com/slowkow/ggrepel) were used to plot the average gene expression. Violin plots (VlnPlot) and feature plots (FeaturePlot) were used to visualize specific gene expressions across clusters and different sample conditions.

### Assay for Transposase-Accessible Chromatin with high-throughput sequencing (ATAC-seq)

WT and ARID1A^−/−^ hESCs were differentiated for 4 days; then, cells were dissociated by Trypsin-EDTA (0.25%). Cells were washed by 1× PBS and resuspended in cold PBS according to ATAC-seq protocol [60]. Briefly, collected cells were lysed in cold lysis buffer (10 mM Tris-HCl, pH 7.4, 10 mM NaCl, 3 mM MgCl2, and 0.1% IGEPAL CA-630) and the nuclei were pelleted and resuspended in Tn5 enzyme and transposase buffer (Illumina Nextera® DNA library preparation kit, cat# FC-121-1030). The Nextera libraries were amplified using the Nextera® PCR master mix and KAPA biosystems HiFi hotstart readymix (cat # NC0295239) successively. AMPure XP beads (Beckman Coulter cat# A63881) were used to purify the transposed DNA and the amplified PCR products. All libraries were sequenced on a 100-cycle paired-end run on an Illumina NOVAseq instrument. The resulting ATAC-seq libraries were sequenced on Illumina NovaSeq 6000 at CMG of Indiana University School of Medicine, and paired-end 50 bp reads were generated. Illumina adapter sequences and low-quality base calls were trimmed off the paired-end reads with Trim Galore v0.4.3 (http://www.bioinformatics.babraham.ac.uk/projects/trim_galore/). The resulting high-quality reads were aligned to the human reference genome hg38 using bowtie2 (version 2.3.2) [61] with parameters “-X 2000 --no-mixed --no-discordant.” Duplicate reads were discarded with Picard (https://broadinstitute.github.io/picard/). Reads mapped to mitochondrial DNA together with low mapping quality reads (MAPQ < 10) were excluded from further analysis. ATAC-seq was conducted in Genomic Core at Indiana University.

### Integrated analysis of ATAC-seq and ChIP-seq results

Bowtie2 [61] was used to align sequencing reads on the human genome (hg38) for both ChIP-seq and ATAC-seq. Low-quality score (Q30) reads and redundant reads due to PCR bias were filtered by SAMtools [62, 63] and Picard MarkDuplicates that were not considered for further analysis. There were two technical replicates for ATAC-seq for both WT and ARID1A^−/−^. Open chromatin regions for each sample were identified first from peak calling of individual ATAC-seq using MACS2 [64] with broad peak option and cutoff of *q* < 0.1. We then constructed a final set of unique regions for differential analysis by merging overlapped open chromatin regions recognized from different samples/conditions. The read abundance of each open region was counted for each individual sample, respectively, by using pyDNase [65]. A software, edgeR [66, 67], was employed to perform differential accessibility analysis on the read counts of each chromatin region for WT and ARID1A^−/−^ samples. Differentially accessible open chromatin regions between WT and ARID1A^−/−^ were determined by specific cutoffs, FDR-adjusted *p* value less than 0.01, and amplitude of fold change (in log scale with base 2) larger than 0.5.

ARID1A binding sites were determined by model-based ChIP-seq data analysis using MACS2 [64]. Peak calling of uniquely mapped ARID1A ChIP-seq reads was performed by comparing with input ChIP-seq. All binding peaks were recognized if their *p* values were less than 0.01 after the Benjamini-Hochberg multiple-test correction. We examined consensus sequences significantly enriched in ARID1A binding peaks. The motif enrichment analysis was performed by using the HOMER (version 4.9.1) [68] command “findMotifsGenome.pl” with the parameter “-size given.” The open chromatin regions detected by ATAC-seq or ARID1A binding sites identified by ARD1A ChIP-seq were linked to specific genes, if they locate either within upstream 10 kb from TSS, or 5′UTR, or exon/intron (gene body) of the genes. The UCSC genome browser custom track was made to visualize differences of chromatin accessibility between WT and ARID1A^−/−^ for selected regions. The read numbers were normalized by total numbers of high-quality reads in each sample, then multiplied by ten millions. The normalized averaged counts for replicates under the same condition were presented in the figures.

### Flow cytometry

Flow cytometry was performed according to our previous publication [69]. Briefly, cells were harvested and dissociated by 0.25% Trypsin-EDTA for 5–10 min at 37 °C. The dissociated single cells were fixed in 4% PFA (diluted with 16% paraformaldehyde (formaldehyde) aqueous solution) for 10 min at room temperature and washed 3 times with 1× PBS. Cells were incubated in blocking PBS buffer containing 2% goat serum and 0.1% saponin. Then cells were incubated in blocking buffer with primary antibody for 1 h at 37 °C, following with secondary antibody staining for 1 h at 37 °C. Flow cytometry analysis was carried out with Accuri C6 flow cytometer (Becton Dickinson), BD LSRII cytometer (Becton Dickinson) and Attune NxT Flow Cytometer (Thermo Fisher Scientific). Data were analyzed by FlowJo (Treestar).

### Immunofluorescence (IF)

For immunocytochemistry, cells were fixed with 4% PFA (diluted from 16% Paraformaldehyde aqueous solution) for 10 min at room temperature. After washing with 1× PBS, cells were blocked for 1 h with 1× PBS blocking buffer containing 2% goat serum (or 5% BSA) and 0.1% saponin. Staining with primary antibodies diluted with blocking buffer was performed for overnight at − 4 °C. Staining with secondary antibodies was performed on the next day, following with nucleus staining with DAPI. Leica DM6B image system was used for imaging.

### Functional enrichment analysis

The functional enrichment analysis, including Gene Ontology (GO), process networks, signaling pathways, and gene interaction networks, was performed on gene sets selected by using Metacore software (Clarivate Analytics). We chose the cutoff *p* < 0.05 to determine the functions significantly overrepresented in genes of our interest.

### Quantification and statistical analysis

For RT-qPCR and flow cytometry data, comparisons between two groups (KO vs. WT) were conducted using an unpaired two-tailed *t* test. All data were presented as mean ± SD from at least three independent experiments. Differences with *p* values less than 0.05 were considered significant. A non-parametric test, Wilcoxon’s signed-rank test, was used to compare the gene expression difference between WT and ARID1A^−/−^ detected by scRNA-seq. Fisher’s test was adopted to determine the statistical significance of difference of ratios of cell numbers between WT and ARID1A^−/−^. We used hypergeometric model to calculate the significance of overlap between ARID1A and REST target genes, and *p* value of enrichment of cardiac or neural genes in both ARID1A and REST targets.

## Supplementary information

**Additional file 1. Fig. S1:** Establishment of ARID1A null H9 hESC line. **Fig. S2** ScRNAseq analysis of undifferentiated WT and ARID1A−/− hESCs. **Fig. S3** Identity of neural cells from ARID1A−/− hESCs. **Fig. S4** ScRNA-seq reveals loss-of-ARID1A represses cardiac but promotes neural differentiation from hESCs. **Fig. S5** Functional enrichment analysis. **Fig. S6** Loss-of-ARID1A promotes neural differentiation under neural differentiation conditions and knockdown of ARID1A promotes cardiac differentiation for hESCs. **Fig. S7** ARID1A affects chromatin accessibility and interacts with other transcriptional factors.

**Additional file 2: Table S1.** scRNA-seq genes expression profiles.

**Additional file 3:** contractile activity videos of differentiated (day 10) WT and ARID1A KO hESCs.

**Additional file 4: Table S2.**. T4 ATAC-seq paired genes.

**Additional file 5: Table S3.** ARID1A ChIP-seq paired genes.

**Additional file 6: Table S4.** Oligonucleotides.

**Additional file 7.** Review history.

## Data Availability

ChIP-seq, ATAC-seq, and single-cell RNA-seq next-generation sequencing data are available in the NCBI GEO, under accession number GSE139343 [70]. ChIP-seq next-generation sequencing data are available in the NCBI GEO, under accession number GSE139260 [71]. ATAC-seq next-generation sequencing data are available in the NCBI GEO, under accession number GSE139329 [72]. Single-cell RNA-seq next-generation sequencing data are available in the NCBI GEO, under accession number GSE139342 [73]. The authors declare that all other data supporting the findings of this study are within the manuscript and its supplementary files.

## References

[1] Srivastava D (2006). Making or breaking the heart: from lineage determination to morphogenesis. Cell.

[2] Francis F, Meyer G, Fallet-Bianco C, Moreno S, Kappeler C, Socorro AC, Tuy FP, Beldjord C, Chelly J (2006). Human disorders of cortical development: from past to present. Eur J Neurosci.

[3] Hoch RV, Rubenstein JL, Pleasure S (2009). Genes and signaling events that establish regional patterning of the mammalian forebrain. Semin Cell Dev Biol.

[4] Morton PD, Ishibashi N, Jonas RA (2017). Neurodevelopmental abnormalities and congenital heart disease: insights into altered brain maturation. Circ Res.

[5] Knirsch W, Mayer KN, Scheer I, Tuura R, Schranz D, Hahn A, Wetterling K, Beck I, Latal B, Reich B (2017). Structural cerebral abnormalities and neurodevelopmental status in single ventricle congenital heart disease before Fontan procedure. Eur J Cardiothorac Surg.

[6] Ueda Y, Okano M, Williams C, Chen T, Georgopoulos K, Li E (2006). Roles for Dnmt3b in mammalian development: a mouse model for the ICF syndrome. Development.

[7] Zaidi S, Choi M, Wakimoto H, Ma L, Jiang J, Overton JD, Romano-Adesman A, Bjornson RD, Breitbart RE, Brown KK (2013). De novo mutations in histone-modifying genes in congenital heart disease. Nature.

[8] Bruneau BG (2010). Chromatin remodeling in heart development. Curr Opin Genet Dev.

[9] Lickert H, Takeuchi JK, Von Both I, Walls JR, McAuliffe F, Adamson SL, Henkelman RM, Wrana JL, Rossant J, Bruneau BG (2004). Baf60c is essential for function of BAF chromatin remodelling complexes in heart development. Nature.

[10] Hang CT, Yang J, Han P, Cheng HL, Shang C, Ashley E, Zhou B, Chang CP (2010). Chromatin regulation by Brg1 underlies heart muscle development and disease. Nature.

[11] Yoo AS, Staahl BT, Chen L, Crabtree GR (2009). MicroRNA-mediated switching of chromatin-remodelling complexes in neural development. Nature.

[12] Weissman B, Knudsen KE (2009). Hijacking the chromatin remodeling machinery: impact of SWI/SNF perturbations in cancer. Cancer Res.

[13] Ronan JL, Wu W, Crabtree GR (2013). From neural development to cognition: unexpected roles for chromatin. Nat Rev Genet.

[14] Alpsoy A, Dykhuizen EC (2018). Glioma tumor suppressor candidate region gene 1 (GLTSCR1) and its paralog GLTSCR1-like form SWI/SNF chromatin remodeling subcomplexes. J Biol Chem.

[15] Chandler RL, Brennan J, Schisler JC, Serber D, Patterson C, Magnuson T (2013). ARID1a-DNA interactions are required for promoter occupancy by SWI/SNF. Mol Cell Biol.

[16] Gao X, Tate P, Hu P, Tjian R, Skarnes WC, Wang Z (2008). ES cell pluripotency and germ-layer formation require the SWI/SNF chromatin remodeling component BAF250a. Proc Natl Acad Sci U S A.

[17] Chandler RL, Magnuson T (2016). The SWI/SNF BAF-A complex is essential for neural crest development. Dev Biol.

[18] Kosho T, Okamoto N, Ohashi H, Tsurusaki Y, Imai Y, Hibi-Ko Y, Kawame H, Homma T, Tanabe S, Kato M (2013). Clinical correlations of mutations affecting six components of the SWI/SNF complex: detailed description of 21 patients and a review of the literature. Am J Med Genet A.

[19] Tsurusaki Y, Okamoto N, Ohashi H, Kosho T, Imai Y, Hibi-Ko Y, Kaname T, Naritomi K, Kawame H, Wakui K (2012). Mutations affecting components of the SWI/SNF complex cause Coffin-Siris syndrome. Nat Genet.

[20] Santen GW, Aten E, Sun Y, Almomani R, Gilissen C, Nielsen M, Kant SG, Snoeck IN, Peeters EA, Hilhorst-Hofstee Y (2012). Mutations in SWI/SNF chromatin remodeling complex gene ARID1B cause Coffin-Siris syndrome. Nat Genet.

[21] Van Houdt JK, Nowakowska BA, Sousa SB, van Schaik BD, Seuntjens E, Avonce N, Sifrim A, Abdul-Rahman OA, van den Boogaard MJ, Bottani A (2012). Heterozygous missense mutations in SMARCA2 cause Nicolaides-Baraitser syndrome. Nat Genet.

[22] Hayashi R, Ishikawa Y, Sasamoto Y, Katori R, Nomura N, Ichikawa T, Araki S, Soma T, Kawasaki S, Sekiguchi K (2016). Co-ordinated ocular development from human iPS cells and recovery of corneal function. Nature.

[23] McCracken KW, Cata EM, Crawford CM, Sinagoga KL, Schumacher M, Rockich BE, Tsai YH, Mayhew CN, Spence JR, Zavros Y, Wells JM (2014). Modelling human development and disease in pluripotent stem-cell-derived gastric organoids. Nature.

[24] Lancaster MA, Renner M, Martin CA, Wenzel D, Bicknell LS, Hurles ME, Homfray T, Penninger JM, Jackson AP, Knoblich JA (2013). Cerebral organoids model human brain development and microcephaly. Nature.

[25] Nishikawa S, Goldstein RA, Nierras CR (2008). The promise of human induced pluripotent stem cells for research and therapy. Nat Rev Mol Cell Biol.

[26] Lin B, Kim J, Li Y, Pan H, Carvajal-Vergara X, Salama G, Cheng T, Li Y, Lo CW, Yang L (2012). High-purity enrichment of functional cardiovascular cells from human iPS cells. Cardiovasc Res.

[27] Li Y, Lin B, Yang L (2015). Comparative transcriptomic analysis of multiple cardiovascular fates from embryonic stem cells predicts novel regulators in human cardiogenesis. Sci Rep.

[28] Butler A, Hoffman P, Smibert P, Papalexi E, Satija R (2018). Integrating single-cell transcriptomic data across different conditions, technologies, and species. Nat Biotechnol.

[29] McCarthy DJ, Campbell KR, Lun AT, Wills QF (2017). Scater: pre-processing, quality control, normalization and visualization of single-cell RNA-seq data in R. Bioinformatics.

[30] Burridge PW, Matsa E, Shukla P, Lin ZC, Churko JM, Ebert AD, Lan F, Diecke S, Huber B, Mordwinkin NM (2014). Chemically defined generation of human cardiomyocytes. Nat Methods.

[31] Guo X, Xu Y, Wang Z, Wu Y, Chen J, Wang G, Lu C, Jia W, Xi J, Zhu S (2018). A Linc1405/Eomes complex promotes cardiac mesoderm specification and cardiogenesis. Cell Stem Cell.

[32] Pfeiffer MJ, Quaranta R, Piccini I, Fell J, Rao J, Ropke A, Seebohm G, Greber B (2018). Cardiogenic programming of human pluripotent stem cells by dose-controlled activation of EOMES. Nat Commun.

[33] Wu SM (2008). Mesp1 at the heart of mesoderm lineage specification. Cell Stem Cell.

[34] Kitajima S, Takagi A, Inoue T, Saga Y (2000). MesP1 and MesP2 are essential for the development of cardiac mesoderm. Development.

[35] Saga Y, Miyagawa-Tomita S, Takagi A, Kitajima S, Miyazaki J, Inoue T (1999). MesP1 is expressed in the heart precursor cells and required for the formation of a single heart tube. Development.

[36] Saga Y, Hata N, Kobayashi S, Magnuson T, Seldin MF, Taketo MM (1996). MesP1: a novel basic helix-loop-helix protein expressed in the nascent mesodermal cells during mouse gastrulation. Development.

[37] King T, Beddington RS, Brown NA (1998). The role of the brachyury gene in heart development and left-right specification in the mouse. Mech Dev.

[38] Schoenherr CJ, Anderson DJ (1995). The neuron-restrictive silencer factor (NRSF): a coordinate repressor of multiple neuron-specific genes. Science.

[39] Lei I, Gao X, Sham MH, Wang Z (2012). SWI/SNF protein component BAF250a regulates cardiac progenitor cell differentiation by modulating chromatin accessibility during second heart field development. J Biol Chem.

[40] Bultman S, Gebuhr T, Yee D, La Mantia C, Nicholson J, Gilliam A, Randazzo F, Metzger D, Chambon P, Crabtree G, Magnuson T (2000). A Brg1 null mutation in the mouse reveals functional differences among mammalian SWI/SNF complexes. Mol Cell.

[41] Klochendler-Yeivin A, Fiette L, Barra J, Muchardt C, Babinet C, Yaniv M (2000). The murine SNF5/INI1 chromatin remodeling factor is essential for embryonic development and tumor suppression. EMBO Rep.

[42] Kim JK, Huh SO, Choi H, Lee KS, Shin D, Lee C, Nam JS, Kim H, Chung H, Lee HW (2001). Srg3, a mouse homolog of yeast SWI3, is essential for early embryogenesis and involved in brain development. Mol Cell Biol.

[43] Lessard J, Wu JI, Ranish JA, Wan M, Winslow MM, Staahl BT, Wu H, Aebersold R, Graef IA, Crabtree GR (2007). An essential switch in subunit composition of a chromatin remodeling complex during neural development. Neuron.

[44] Wu JI, Lessard J, Olave IA, Qiu Z, Ghosh A, Graef IA, Crabtree GR (2007). Regulation of dendritic development by neuron-specific chromatin remodeling complexes. Neuron.

[45] Zaidi SK, Frietze SE, Gordon JA, Heath JL, Messier T, Hong D, Boyd JR, Kang M, Imbalzano AN, Lian JB, et al. Bivalent epigenetic control of oncofetal gene expression in cancer. Mol Cell Biol. 2017;37:e00352–17. 10.1128/MCB.00352-17 PMC5686582 28923849

[46] Voigt P, Tee WW, Reinberg D (2013). A double take on bivalent promoters. Genes Dev.

[47] Bernstein BE, Mikkelsen TS, Xie X, Kamal M, Huebert DJ, Cuff J, Fry B, Meissner A, Wernig M, Plath K (2006). A bivalent chromatin structure marks key developmental genes in embryonic stem cells. Cell.

[48] Junghans D, Herzog S (2018). Cnn3 regulates neural tube morphogenesis and neuronal stem cell properties. FEBS J.

[49] Ishibashi M, Ang SL, Shiota K, Nakanishi S, Kageyama R, Guillemot F (1995). Targeted disruption of mammalian hairy and enhancer of split homolog-1 (HES-1) leads to up-regulation of neural helix-loop-helix factors, premature neurogenesis, and severe neural tube defects. Genes Dev.

[50] Copp AJ, Greene ND (2010). Genetics and development of neural tube defects. J Pathol.

[51] Chen ZF, Paquette AJ, Anderson DJ (1998). NRSF/REST is required in vivo for repression of multiple neuronal target genes during embryogenesis. Nat Genet.

[52] Battaglioli E, Andres ME, Rose DW, Chenoweth JG, Rosenfeld MG, Anderson ME, Mandel G (2002). REST repression of neuronal genes requires components of the hSWI.SNF complex. J Biol Chem.

[53] Chong JA, Tapia-Ramirez J, Kim S, Toledo-Aral JJ, Zheng Y, Boutros MC, Altshuller YM, Frohman MA, Kraner SD, Mandel G (1995). REST: a mammalian silencer protein that restricts sodium channel gene expression to neurons. Cell.

[54] Ooi L, Belyaev ND, Miyake K, Wood IC, Buckley NJ (2006). BRG1 chromatin remodeling activity is required for efficient chromatin binding by repressor element 1-silencing transcription factor (REST) and facilitates REST-mediated repression. J Biol Chem.

[55] Tuoc TC, Boretius S, Sansom SN, Pitulescu ME, Frahm J, Livesey FJ, Stoykova A (2013). Chromatin regulation by BAF170 controls cerebral cortical size and thickness. Dev Cell.

[56] Ludwig TE, Bergendahl V, Levenstein ME, Yu J, Probasco MD, Thomson JA (2006). Feeder-independent culture of human embryonic stem cells. Nat Methods.

[57] Ludwig TE, Levenstein ME, Jones JM, Berggren WT, Mitchen ER, Frane JL, Crandall LJ, Daigh CA, Conard KR, Piekarczyk MS (2006). Derivation of human embryonic stem cells in defined conditions. Nat Biotechnol.

[58] Sanjana NE, Shalem O, Zhang F (2014). Improved vectors and genome-wide libraries for CRISPR screening. Nat Methods.

[59] Peltier HJ, Latham GJ (2008). Normalization of microRNA expression levels in quantitative RT-PCR assays: identification of suitable reference RNA targets in normal and cancerous human solid tissues. RNA.

[60] Buenrostro JD, Giresi PG, Zaba LC, Chang HY, Greenleaf WJ (2013). Transposition of native chromatin for fast and sensitive epigenomic profiling of open chromatin, DNA-binding proteins and nucleosome position. Nat Methods.

[61] Langmead B, Salzberg SL (2012). Fast gapped-read alignment with bowtie 2. Nat Methods.

[62] Li H, Handsaker B, Wysoker A, Fennell T, Ruan J, Homer N, Marth G, Abecasis G, Durbin R, Genome Project Data Processing S (2009). The sequence alignment/map format and SAMtools. Bioinformatics.

[63] Li H (2011). A statistical framework for SNP calling, mutation discovery, association mapping and population genetical parameter estimation from sequencing data. Bioinformatics.

[64] Zhang Y, Liu T, Meyer CA, Eeckhoute J, Johnson DS, Bernstein BE, Nusbaum C, Myers RM, Brown M, Li W, Liu XS (2008). Model-based analysis of ChIP-Seq (MACS). Genome Biol.

[65] Piper J, Elze MC, Cauchy P, Cockerill PN, Bonifer C, Ott S (2013). Wellington: a novel method for the accurate identification of digital genomic footprints from DNase-seq data. Nucleic Acids Res.

[66] Robinson MD, McCarthy DJ, Smyth GK (2010). edgeR: a Bioconductor package for differential expression analysis of digital gene expression data. Bioinformatics.

[67] McCarthy DJ, Chen Y, Smyth GK (2012). Differential expression analysis of multifactor RNA-Seq experiments with respect to biological variation. Nucleic Acids Res.

[68] Heinz S, Benner C, Spann N, Bertolino E, Lin YC, Laslo P, Cheng JX, Murre C, Singh H, Glass CK (2010). Simple combinations of lineage-determining transcription factors prime cis-regulatory elements required for macrophage and B cell identities. Mol Cell.

[69] Lu TY, Lin B, Li Y, Arora A, Han L, Cui C, Coronnello C, Sheng Y, Benos PV, Yang L (2013). Overexpression of microRNA-1 promotes cardiomyocyte commitment from human cardiovascular progenitors via suppressing WNT and FGF signaling pathways. J Mol Cell Cardiol.

[70] Liu J, Liu S, Gao H, Han L, Chu X, Sheng Y, Wang Y, Shou W, Liu Y, Wan J, Yang L. Genome-wide studies reveal the essential and opposite roles of ARID1A in controlling human cardiogenesis and neurogenesis from pluripotent stem cells. All ChIP-seq, ATAC-seq, single-cell RNA-seq next-generation sequencing libraries presented in this publication. Gene Expression Omnibus. 2020. https://www.ncbi.nlm.nih.gov/geo/query/acc.cgi?acc=GSE139343. Accessed 2020. 10.1186/s13059-020-02082-4 PMC7350744 32646524

[71] Liu J, Liu S, Gao H, Han L, Chu X, Sheng Y, Wang Y, Shou W, Liu Y, Wan J, Yang L. Genome-wide studies reveal the essential and opposite roles of ARID1A in controlling human cardiogenesis and neurogenesis from pluripotent stem cells. All ChIP-seq next-generation sequencing libraries presented in this publication. Gene Expression Omnibus. 2020. https://www.ncbi.nlm.nih.gov/geo/query/acc.cgi?acc=GSE139260. Accessed 2020. 10.1186/s13059-020-02082-4 PMC7350744 32646524

[72] Liu J, Liu S, Gao H, Han L, Chu X, Sheng Y, Wang Y, Shou W, Liu Y, Wan J, Yang L. Genome-wide studies reveal the essential and opposite roles of ARID1A in controlling human cardiogenesis and neurogenesis from pluripotent stem cells. All ATAC-seq next-generation sequencing libraries presented in this publication. Gene Expression Omnibus. 2020. https://www.ncbi.nlm.nih.gov/geo/query/acc.cgi?acc=GSE139329. Accessed 2020. 10.1186/s13059-020-02082-4 PMC7350744 32646524

[73] Liu J, Liu S, Gao H, Han L, Chu X, Sheng Y, Wang Y, Shou W, Liu Y, Wan J, Yang L. Genome-wide studies reveal the essential and opposite roles of ARID1A in controlling human cardiogenesis and neurogenesis from pluripotent stem cells. All single-cell RNA-seq next-generation sequencing libraries presented in this publication. Gene Expression Omnibus. 2020. https://www.ncbi.nlm.nih.gov/geo/query/acc.cgi?acc=GSE139342. Accessed 2020. 10.1186/s13059-020-02082-4 PMC7350744 32646524

