# Supplementary material for: Genome-wide studies reveal the essential and opposite roles of ARID1A in controlling human cardiogenesis and neurogenesis from pluripotent stem cells
Source: Genome Biol. 2020 Jul 9;21:169. doi: 10.1186/s13059-020-02082-4 (PMC7350744; doi:10.1186/s13059-020-02082-4)
Supplement: Supplementary file 1 — Additional file 1. Fig. S1: Establishment of ARID1A null H9 hESC line. Fig. S2 ScRNAseq analysis of undifferentiated WT and ARID1A−/− hESCs. Fig. S3 Identity of neural cells from ARID1A−/− hESCs. Fig. S4 ScRNA-seq reveals loss-of-ARID1A represses cardiac but promotes neural differentiation from hESCs. Fig. S5 Functional enrichment analysis. Fig. S6 Loss-of-ARID1A promotes neural differentiation under neural differentiation conditions and knockdown of ARID1A promotes cardiac differentiation for hESCs. Fig. S7 ARID1A affects chromatin accessibility and interacts with other transcriptional factors. [file 13059_2020_2082_MOESM1_ESM.pdf]

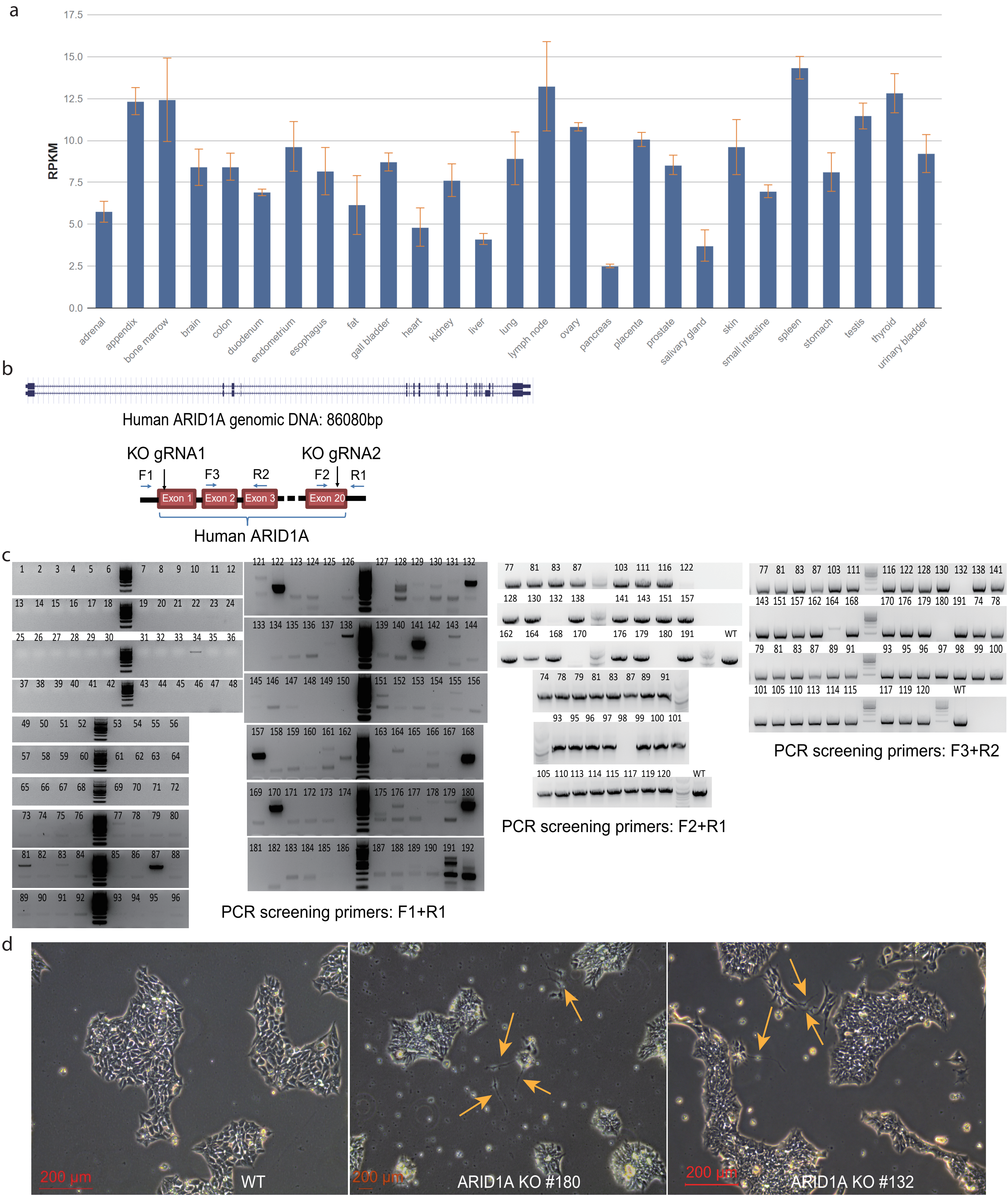

Fig. S1 Establishment of ARID1A null H9 hESC line.
a ARID1A expression in different human samples from NCBI database.
b ARID1A was completely knocked out from hESC H9 cell line by using dual gRNAs mediated by CRISPR/Cas9 technology.
c Identification of ARID1A knockout (KO) hESC clones by using PCR.
d WT and ARID1A KO hESCs cultured in mTesR medium. WT hESCs show normal stem cell morphology.
ARID1A KO hESCs give rise to small spontaneous differentiated cell clusters (yellow arrows).

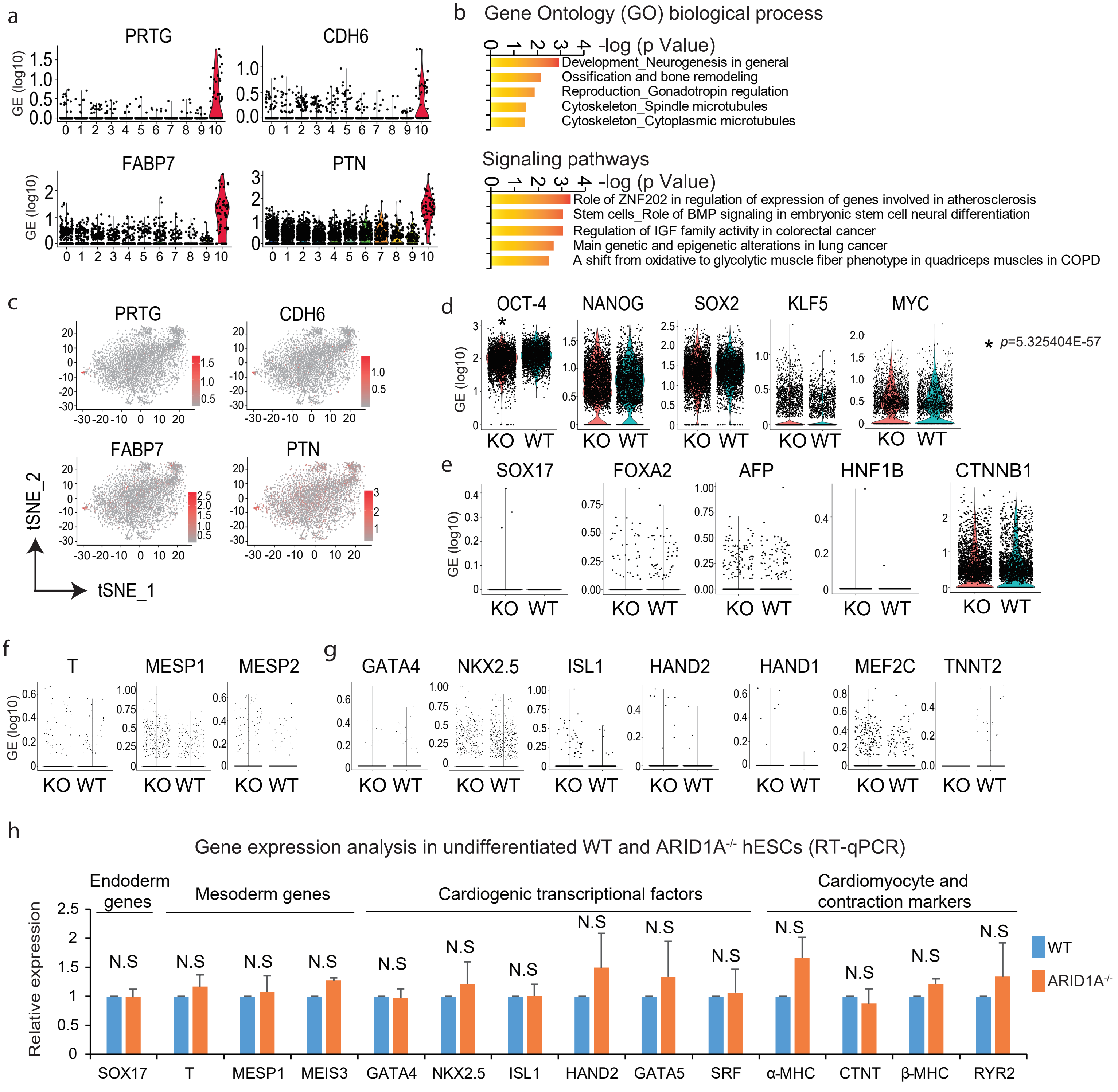

Fig. S2 ScRNAseq analysis of undifferentiated WT and ARID1A-/- hESCs.

a Violin plots analysis of neural markers in each cluster.

b GO biological process (top) and signaling pathways (bottom) analysis of upregulated genes (KO vs. WT) in the cluster 10. Analysis was performed by Metacore software.

c Feature plots of neural markers in integrative WT and KO scRNA-seq data.

d Violin plots analysis of pluripotency markers in WT and KO hESCs. * p=5.325404E-57 (Nonparametric Wilcoxon signed-rank test).

e Violin plots analysis of endodermal markers in WT and KO hESCs. All p values were larger than 0.05 (KO vs. WT), by Nonparametric Wilcoxon signed-rank test.

f Violin plots analysis of early mesodermal markers in WT and KO hESCs. All p values were larger than 0.05 (KO vs. WT), by Nonparametric Wilcoxon signed-rank test.

g Violin plots analysis of cardiac and cardiomyocyte markers in WT and KO hESCs. All p values were larger than 0.05 (KO vs. WT), by Nonparametric Wilcoxon signed-rank test.

h RT-qPCR verification showing gene expression in undifferentiated WT and ARID1A$^{-/-}$ hESCs. N.S, no significance (ARID1A$^{-/-}$ vs. WT), by unpaired two-tailed t-test.

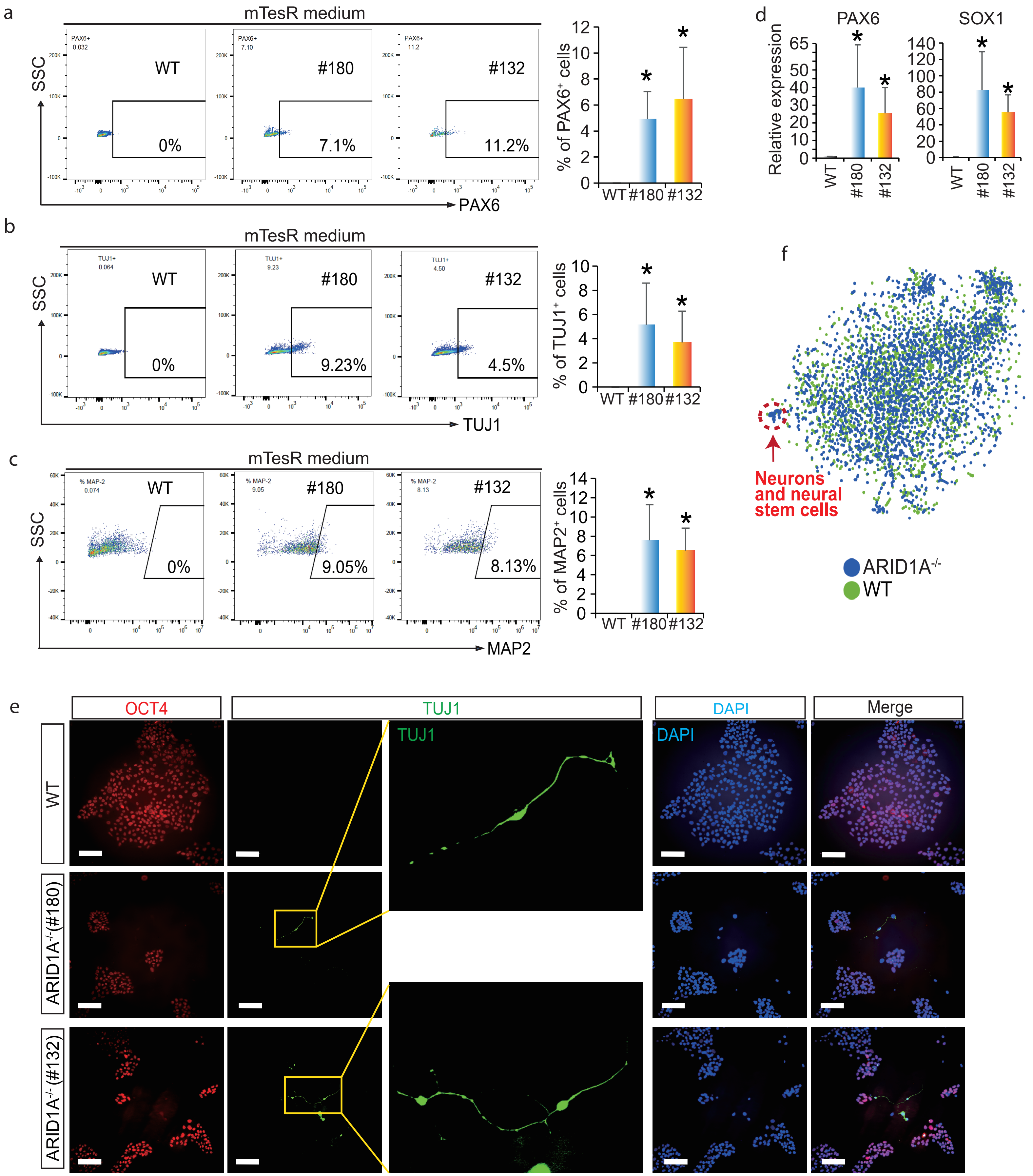

Fig. S3 Identity of neural cells from ARID1A$^{-/-}$ hESCs.
a Quantification of the ratio of PAX6^{+} cells derived from undifferentiated WT and KO hESCs (#180, #132) by flow cytometry.
b Quantification of the ratio of TUJ1^{+} cells derived from undifferentiated WT and KO hESCs (#180, #132) by flow cytometry.
c Quantification of the ratio of MAP2^{+} cells derived from undifferentiated WT and KO hESCs (#180, #132) by flow cytometry.
d RT-qPCR showing the expression levels of PAX6 and SOX1 from undifferentiated WT and KO hESCs (#180, #132).
e Immunofluorescence analysis of pluripotency marker OCT4 and neuron marker TUJ1 in WT and KO hESCs (#180, #132). Scale bar, 100 μm.
f Interactive analysis of scRNA-seq datasets from undifferentiated WT and KO hESCs.
All bars are shown as mean ± SD. n=3, *p < 0.05 (an unpaired two-tailed t-test with Welch's correction).

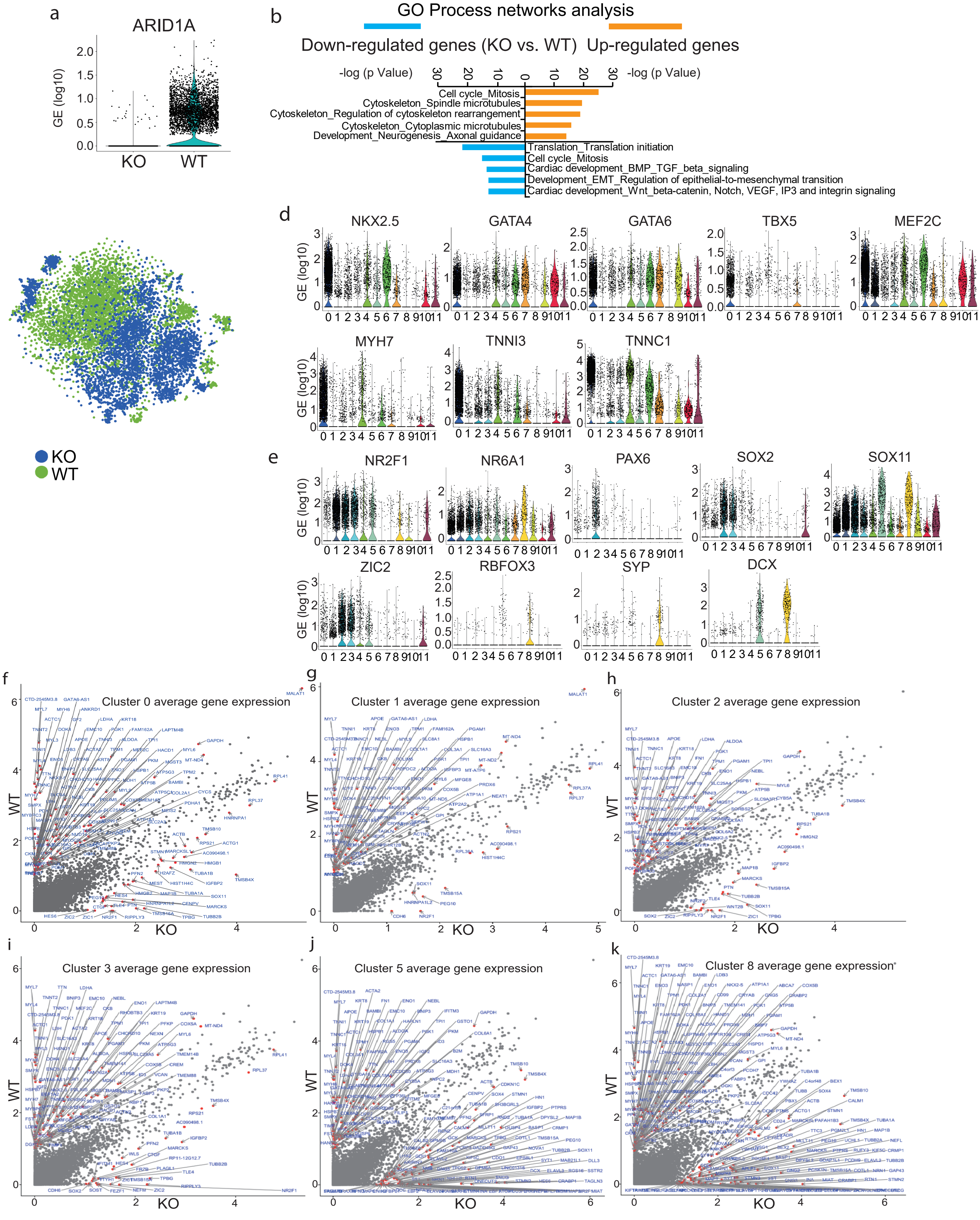

Fig. S4 ScRNA-seq reveals loss-of-ARID1A represses cardiac but promotes neural differentiation from hESCs.
a ARID1A expressions in WT and KO hESC-derived cells by scRNA-seq.
b GO process networks analysis of down-regulated genes and up-regulated genes (KO vs. WT) by Metacore software.
c Integrative analysis of scRNA-seq data from differentiated WT and KO cells. Blue blots showed the KO cells and green blots showed the WT cells.
d Violin plots analysis of cardiogenic gene expressions in each cluster shown with integrative WT and KO scRNA-seq dataset.
e Violin plots analysis of neurogenic gene expressions in each cluster shown with integrative WT and KO scRNA-seq dataset.
f-k Differential gene expression analyses in cluster 0 (f), cluster 1 (g), cluster 2 (h), cluster 3 (i), cluster 5 (j) and cluster 8 (k).

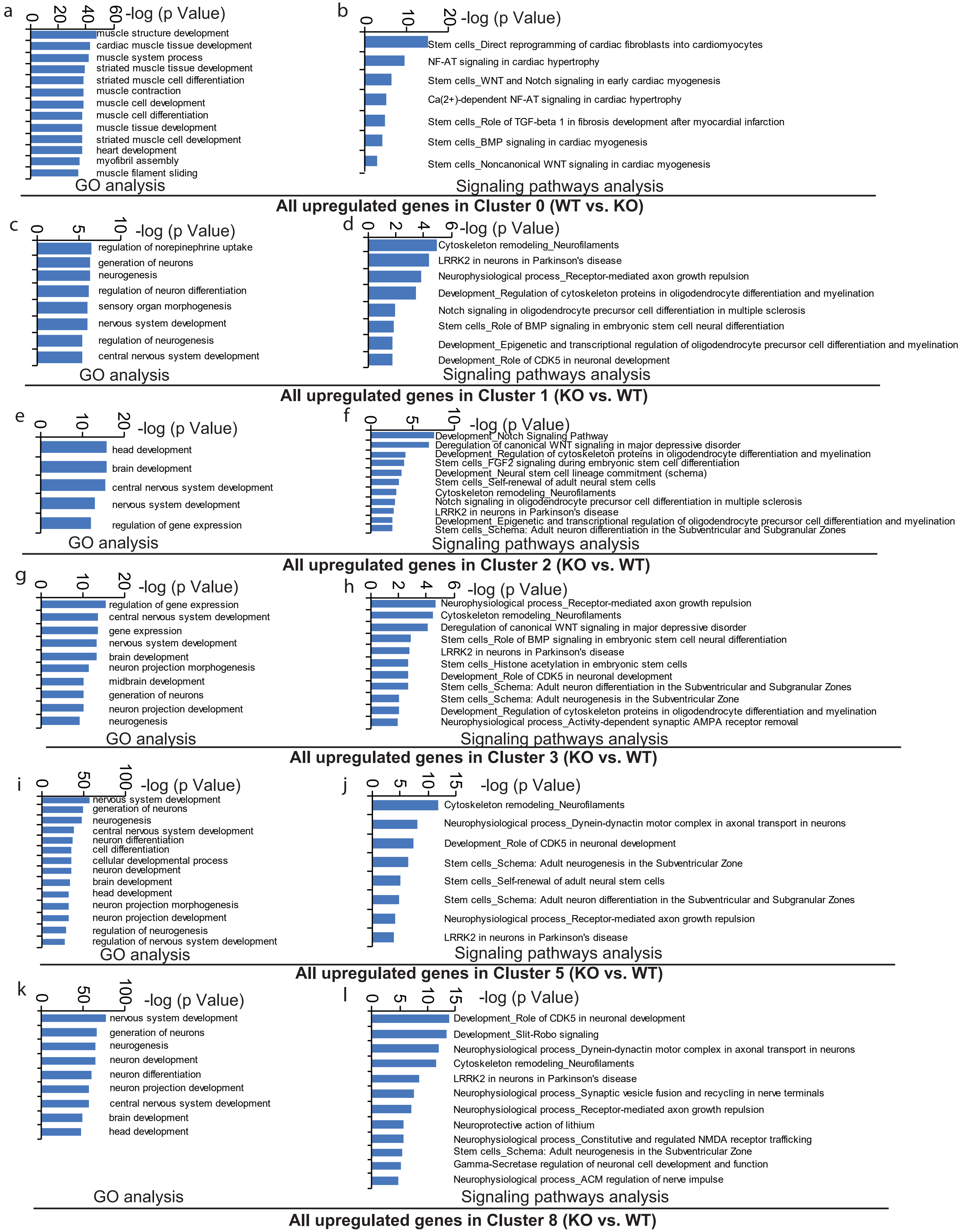

Fig. S5 Functional enrichment analysis.
a-b GO biological process (left) and signaling pathways (right) analysis of all upregulated genes (WT vs. KO) in cluster 0.
c-l GO biological process (left) and signaling pathways (right) analysis of all upregulated genes (KO vs. WT) in Cluster (c-d) 1, Cluster 2 (e-f), Cluster 3 (g-h), Cluster 5 (i-j) and Cluster 8 (k-l).

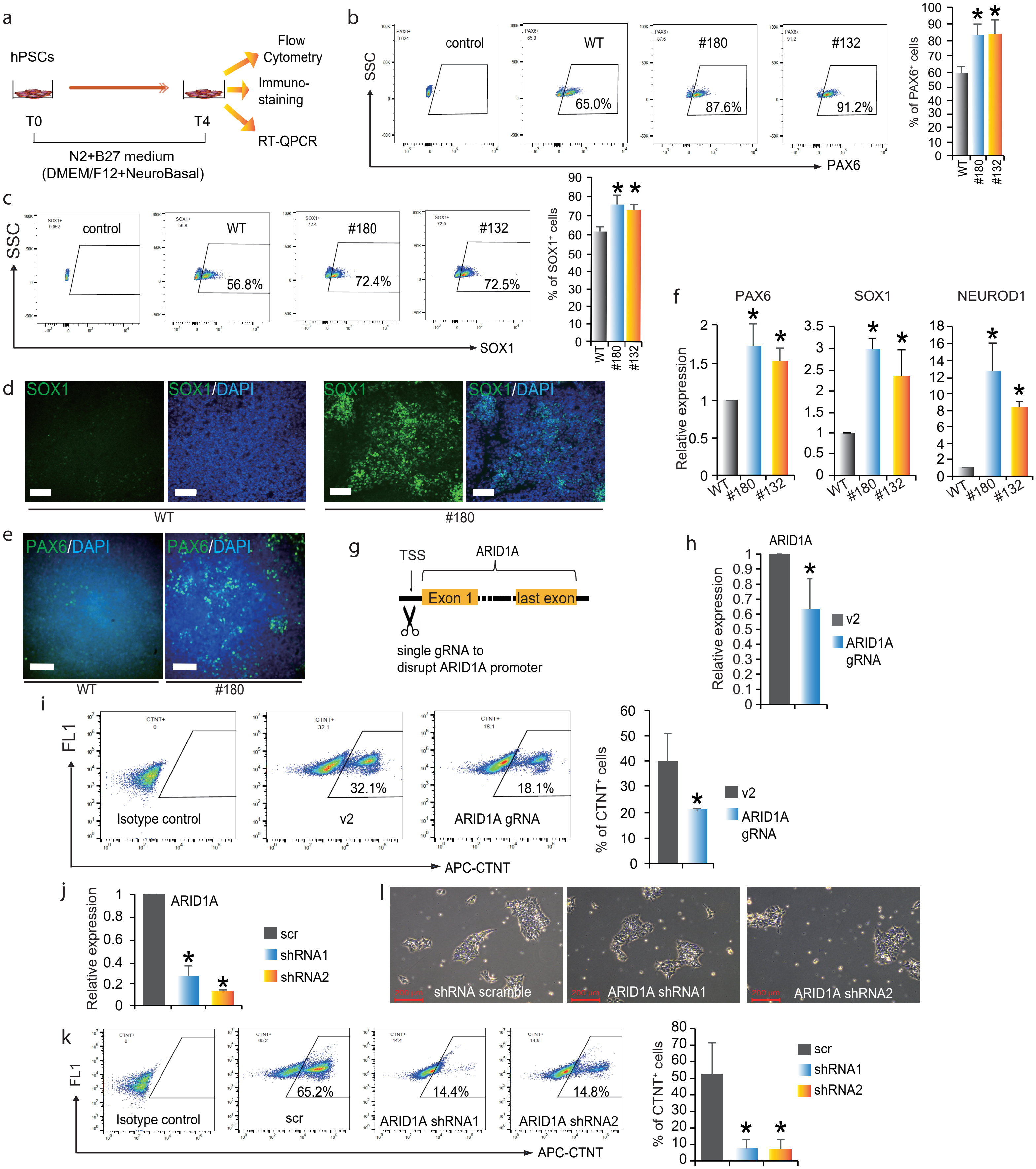

Fig. S6 Loss-of-ARID1A promotes neural differentiation under neural differentiation conditions and knockdown of ARID1A promotes cardiac differentiation for hESCs

a Strategy for neural differentiation of WT and ARID1A$^{-/-}$ hESCs.

b-c Flow cytometry data showing increased percentages of PAX6^{+} (b) and SOX1^{+} (c) cells derived from ARID1A KO hESCs (#180, #132) post specific neural differentiation when compared to that of WT hESCs.

d-e Loss-of-ARID1A (#180) increased the differentiation of SOX1^{+} (d) and PAX6^{+} (e) cells after neural differentiation than WT hESCs by immunostaining. Scale bar, 100 μm.

f QRT-PCR analysis of PAX6, SOX1 and NEUROD1 mRNA expression levels post neural differentiation.

g Human ARID1A promoter was disrupted by single gRNA (ARID1A gRNA) targeting TSS.

h ARID1A mRNA expression was decreased by ARID1A TSS-gRNA in hESCs.

i Flow cytometry data showing knockdown of ARID1A decreased percentage of CTNT^{+} cells compared to that of WT, after 10 days of cardiac differentiation.

j ARID1A expression levels were decreased by two shRNAs in H9 hESCs.

k ARID1A shRNAs decreased percentage of CTNT+ cells after 10 days of cardiac differentiation.

l Both of WT and ARID1Aknockdown hESCs were cultured in mTesR medium, showing normal stem cell morphology without any visible differentiated cells. Scale bar, 200 μm.

All bars are shown as mean ± SD. n=3, $*p < 0.05$ (an unpaired two-tailed t-test).

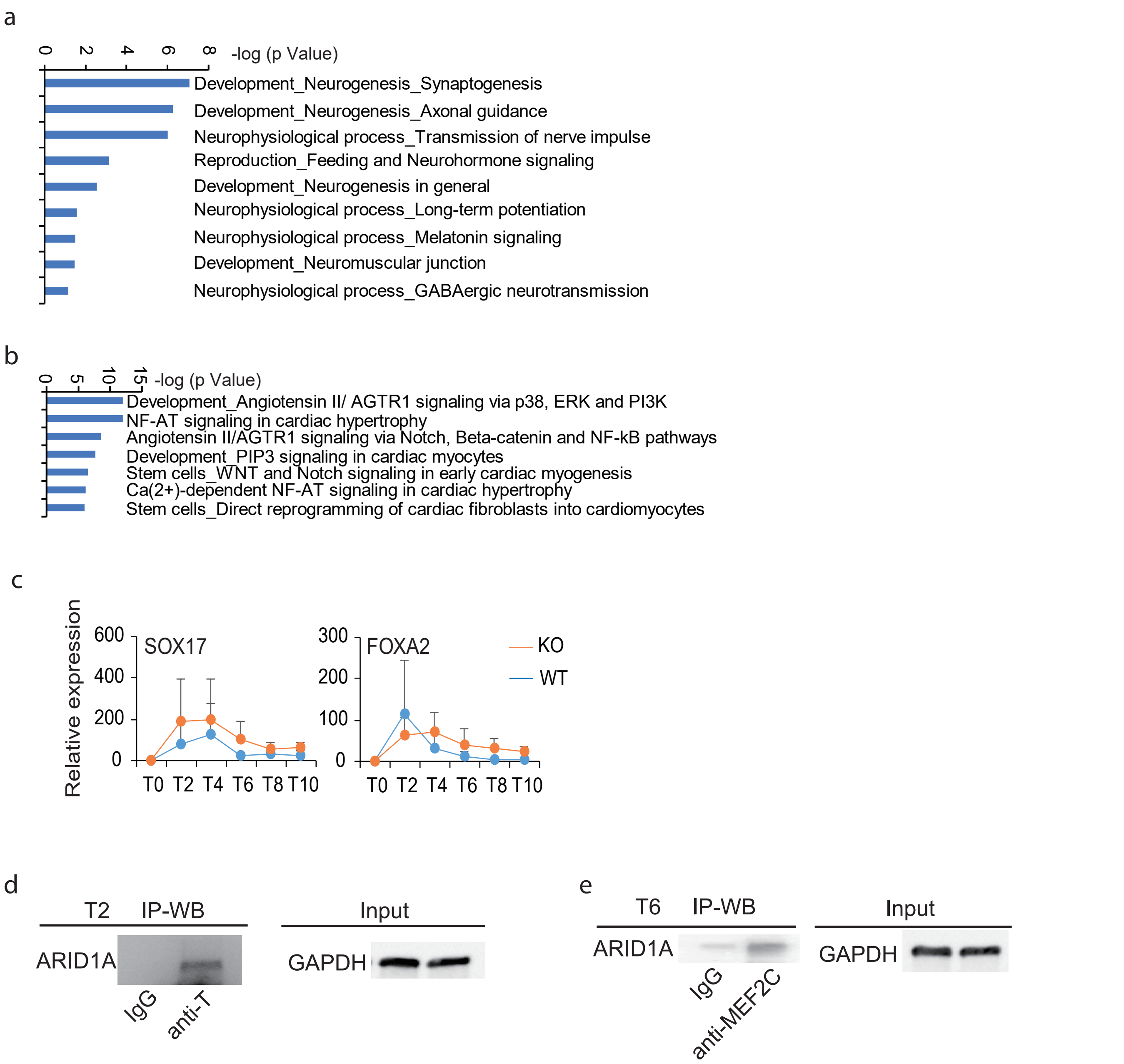

Fig. S7 ARID1A affects chromatin accessibility and interacts with other transcriptional factors.

a Process networks analysis of genes with increased chromatin accessibilities (WT vs. KO) by Metacore.

b Signaling pathways analysis of genes with decreased chromatin accessibilities (WT vs. KO) by Metacore.

c Temporal mRNA expression profiles of endodermal markers SOX17 and FOXA2 during cardiac differentiation. WT and KO hESCs were differentiated from day 0 (T0) to day 10 (T10), and RNAs were collected every 2 days. All bars are shown as mean ± SD. n=3. Unpaired two-tailed t-test with Welch's correction.

d Co-IP validation of interaction of T and ARID1A by using anti-T antibody to pull down ARID1A after 2 days (T2) of cardiac differentiation.

e Co-IP validation of interaction of MEF2C and ARID1A by using anti-MEF2C antibody to pull down ARID1A after 6 days (T6) of cardiac differentiation.
