# Supplementary material for: Genome-wide studies reveal the essential and opposite roles of ARID1A in controlling human cardiogenesis and neurogenesis from pluripotent stem cells
Source: Genome Biol. 2020 Jul 9;21:169. doi: 10.1186/s13059-020-02082-4 (PMC7350744; doi:10.1186/s13059-020-02082-4)
Supplement: Supplementary file 7 — Additional file 7. Review history. [file 13059_2020_2082_MOESM7_ESM.docx]

Review History

**First round of review**

**Reviewer 1**

**Are you able to assess all statistics in the manuscript, including the appropriateness of statistical tests used?**

No, I do not feel adequately qualified to assess the statistics.

**Comments to author:**

This manuscript titled "Genome-wide studies reveal the essential and opposite roles of ARID1A in controlling human cardiogenesis and neurogenesis from pluripotent stem cells" by Liu et al., describes the role of chromatin remodeler protein ARID1A in regulating early human neural and cardiac differerntiation using a directed differentiation protocols. They use different techniques to interrogate the system and claim that ARID1A has two different roles in activating cardiogenesis in a chromatin remodeling dependent manner while repressing neurogenesis in a chromatin remodeling independent manner. They identify MEF2C and T as the transcription factors that drive initiation of cardiac mesoderm and cardiogenesis and show that ARID1A also interacts with neuronal repressor REST to prevent neurogenesis. This paper attempts to answer a very fundamental question in the developmental biology field as to how cells make different lineage decision but fails to answer it, largely as the authors start with a cell line that spontaneously differentiates from the beginning, indicating a fundamental defect in maintenance of pluripotency. The directed differentiation experiments fail to convince that ARID1A

Specific comments:

1. A very small percent of The ARID1A-/- cells spontaneously differentiate to neurons and express pan neuronal marker TUJ1 as well as more mature neuron marker MAP2 in ES cell culture conditions. The authors make the argument this is due to loss of ARID1A. Why only a small percentage of cells? If it is really ARID1A dependent shouldn't this be observed homogenously in most if not all of the cells? What prevents most other ARID1A-/- cells to not to undergo this kind of differentiation? Could the authors sub-clone their mutant colonies to have more homogenous population? The spontaneous loss of pluripotency towards a variety of cell types leads to a very different conclusion.

2. The authors state in the abstract that "cardiac differentiation from ARID1A-/- hESCs was fully suppressed. ARID1A KO only decreased cardiac differentiation efficiency and didn't fully abolish it. In fact even in their directed differentiation system they achieve only 50% cardiomyocytes, and in the absence of ARID1A it goes down to 20%. This is hardly a cardiac to neural switch, especially as in WT differentiations the authors report a small percentage of neural cells. At best this indicates that ARID1A tilts the balance from mesoderm to ectoderm. Incidentally, the text describing Fig 3 is very difficult to follow and doesn't well represent the data.

3. ARID1a loss in-vivo mice die E6.5 for lack of primitive streak formation, how the authors reconcile this? Does Over expression of ARID1A promote cardiac differentiation efficiency?

4. Dissecting role of ARID1a affecting transcription by chromatin remodeling (cardiac) and chromatin remodeling independent (neural) is critical and could have given important insights into ARID1a role but is not addressed.

5. Figure 1B and 1C needs more clarification. Are they GO biological process, molecular function or cellular components or anything else?

6. Figure 1E: Many subunits shown are not major SWI/SNF complex components. Authors need to change line 10 wordings. Showing all the major components of SWI/SNF and how their expression changes across these five different cell type will be useful to the readers.

7. From Figure 1, it cannot be claimed that "ARID1A is involved in cardiac fate commitment from ESCs" as the authors do. It says at best that ARID1A expression is increased from hESCs to multipotent cardiovascular progenitor cells and cardiomyocytes and should be stated as such.

8. Figure 2D: Is it normal to have ~ 10% of WT cells express mature neuron marker MAP2? Does this means the culture condition employed here produces spontaneous differentiations of even WT cells?

9. There are inconsistencies that the authors need to address between Figure 2D, and Figure 2K in MAP2 expression data from single cell RNASeq and FACS in WT and ARID1A-/- cells. They show different percentages.

10. Supplementary Figure 2F: It appears that there is an increase of pre-cardiac and cardiac mesoderm markers EOMES and MESP1 in ARID1a-/- KO cells as well. Does this mean the KO cells are spontaneously differentiating to express these mesodermal markers, although to a lower extent compared to the neural markers?

11. Figure 3L: Why do WT cells shows mature neurons in a cardiac differentiation system? What is the cardiac differentiation efficiency? The authors need to explains this? Also they need to discuss the lack of endothelial cells and fibroblast in ARID1A-/- cells and need to comment if ARID1A facilitates differentiation of endothelial and fibroblasts cells.

12. Figure 5: It would be useful to perform ATAC-seq on the hESCs as spontaneous neural differentiation is observed in these cells. This would also add insights if ARID1A acts in the ES cell stage to alter the chromatin landscape of the cell in order to facilitate lineage specific gene expression programs.

13. Figure 5E: y-axis scale would be necessary as it is difficult to see if KO has high chromatin accessibility than WT.

14. Figure 5E and F: While authors claim that there is increase in chromatin accessibility at the promoters of ZIC1, LHX5, FABP7 and MAP2, in reality the increase is very minor and WT tracks show almost similar chromatin accessibility. How similar chromatin accessibility gives rise to such significant differences in gene expression (Figure 5F) is not at all clear and needs more explanation. With the same logic, the repression of cardiac genes in ARID1A-/- mutants (Figure 5 O)could not be explained based on the chromatin accessibility patterns. Moreover, for Mesp1 and T the decreased chromatin accessibility does not result in change in gene expression. Thus it is difficult to understand if the changes in ARID1A mediated chromatin accessibility cause the changes in gene expression.

15. Figure 6: These ARID1A ChIPs were carried out in hESCs and shows that ARID1A occupies many neural genes promoters as well as cardiac promoters. Why does ARID1A occupy only cardiac and neural promoters at this stage? Is it guarding the fate of these cells only to these two differentiation lineages? How many total ARID1A peaks detected? How many of them occupy neural genes and how many of them occupy cardiac genes? Is it an independent function of ARID1A or of BAF complexes in general? This should be compared to known data. Most importantly what about all other processes that happens in hESCs, or does ARID1A function specifically and only in cardiac and neural processes? These questions needs to be addressed.

Minor:

1. line 5 : neural crescent cells should be neural crest cells

2. line 51 : Ariad1a typo needs to be fixed

**Reviewer 2**

**Are you able to assess all statistics in the manuscript, including the appropriateness of statistical tests used?**

Yes, and I have assessed the statistics in my report.

**Comments to author:**

Liu et al. hypothesize that distinct ARID1A-dependent chromatin remodeling mechanisms govern cardiogenesis and neurogenesis. ARID1A has known roles in cardiac and neural development in vivo. The manuscript utilizes hESC differentiation and single-cell sequencing approaches to address the mechanism of ARID1A-SWI/SNF chromatin remodeling in cardiac versus neural lineage differentiation. Comparisons with ARID1A binding (via ChIPseq) and ARID1A-dependent chromatin accessibility changes (via ATACseq) are provided. This is manuscript is suitable for Genome Biology readership. However, it's very rough around the edges and the data presented lack statistical rigor. As a result, many of the conclusions raised are difficult to interpret. This makes it hard to both properly evaluate and judge the novelty of the findings in the manuscript. Specific comments are listed below.

Comments:

*	Please provide a reviewer token to the GEO submission. Please include a supplemental table with descriptions of the samples submitted and number of replicates performed.

*	Figure panels should be introduced alphabetically in the text.

*	Genomic statements are frequently made with only visual verification from plots. Increase use of statistical tests to backup genomic findings throughout manuscript.

*	Detailed descriptions of the methodology and data analysis are required. Please provide descriptions of the statistical tests used.

*	Please make sure all of the text in figures are legible, or remove if unnecessary. Plot text is frequently too small to read. Several figure panels show text that impossible to read.

*	Frequent grammatical, syntax and spelling errors are found throughout the manuscript and should be corrected. Please have a professional editor review the manuscript.

*	Refer to SWI/SNF as SWI/SNF chromatin remodeling complex rather than epigenetic complex.

*	Student's t-test should only be used with normally distributed data with similar variance between groups. Consider using a nonparametric test for certain analyses where high sampling variance is observed.

*	In the abstract, it is stated that, 'ARID1A recruited T and MEF2C to cadiogenic genes,' but there's no data supporting these conclusions. Either remove these concluding statements or include T or MEF2C ChIPseq or ChIPseq data comparisons.

*	It is not stated how ATAC-seq and ChIP-seq peaks were functionally linked to genes. It is assumed this is based on proximity to gene, but it is not clear whether this is within a certain distance to gene TSS or over gene bodies, etc. This information is not found within the manuscript and should be included.

*	In the results section, 'Therefore, scRNA-seq results demonstrate that loss-of-ARID1A in hESCs exclusively increases transcription of neural-associated marker genes and induces spontaneous neural differentiation, indicating ARID1A plays an important role in repressing neural commitment from hESCs' is a bold statement that is only backed by a few representative cell-marker genes. A more quantitative analysis should be performed to support this claim, such as enrichment tests for cell type gene signatures in all clusters, then can compare observed/expected gene set enrichment ratios. Or, compare relative mean expression values for all genes in a cell type signature between clusters, etc.

*	Number of ATAC-seq replicates for each condition are not stated. Since edgeR was used to calculate differential accessibility, at least 2 replicates are inferred, but this n should be specified. Also, please state what normalized values were used for differential accessibility analysis. Further, 'We then constructed a final set of unique regions by merging overlapped open chromatin regions recognized from different samples/conditions' is vague and should be more specific. Were MACS2 peaks firstly intersected between biological replicates, then taken the union between conditions? "Merged overlapping open chromatin regions" are assumed to be the input for edgeR analysis, but please confirm this in the methods section.

*	Fig. 1E - legend states 'Genes expression profiles of SWI/SNF subunits in different cardiovascular lineages by whole transcriptome sequencing.' Then proceeds to show expression data of ARID1A, ARID1B, and 7 genes encoding non-SWI/SNF ARID domain containing proteins, and SMARCA2 / SMARCC2. The non-SWI/SNF ARID subunits should be omitted from this analysis since, and potentially include other SWI/SNF subunits, like SMARCA4.

*	Fig. 1F - RT-PCR analysis shows only a modest, 1 log2 fold increase in mRNA expression for ARID1A during differentiation. This modest increase may not result in altered protein expression and should be validated by a protein-based assay such as WB.

*	Fig. 1K - the difference between red and white arrows, if any, is not clear. Choose one color if no distinction.

*	Fig. 2B-C - Need to apply a statistical test to quantitatively support these claims, such as wilcox test.

*	Fig. 2D - Again, a statistical test of proportions like Fisher's can better support this claim

*	Fig. 2I - while this is a nice visual, it is redundant with Fig. 2G and should be moved to supplemental

*	Supp. Fig. 2D - Text states " Loss-of-ARID1A did not affect the expression of pluripotency... genes." but POUF51 and SOX2 appear shifted in expression, potentially to a statistically significant degree. Please include summary statistics for any plots shown like this.

*	Fig. 3C-D - same comments as Fig. 2B-C

*	Fig. 3I - same comments as Fig. 2I

*	Supplementary Fig. 4 panels G-K are not referenced in the text.

*	Fig. 4G-J - the difference between panels G and H is not clear. Likewise, for panels I and J.

*	Supp. Fig. 6G and 6K are unnecessary; constructs should be omitted and referenced instead.

*	Fig. 5E - genomic tracks of ATAC-seq data should have y-axis and data type specified in legend. There is currently no information provided. Are these bedgraphs of normalized ATAC signal per bp? MACS2 enrichment score? etc. The ChIP-seq tracks e.g. in Fig. 6F have a y-axis, so something similar should be provided for these ATAC-seq tracks.

*	Fig. 6F, 6I - 'Example views of ARID1A occupancies...' should be more clear. Like in Fig. 5E, please state what data are being shown. Reads per million per base? etc.

*	In Fig. 7I-K and Suppl. Fig. 7, the Co-IP data do not include proper Input controls. The input fractions should be probed the antibodies used to detect interacting proteins.

*	While the FIG. 7 ARID1A vs. REST ChIP-seq comparisons provide support for the model, there's no comparisons between ARID1A and T or MEF2C.

**Reviewer 3**

**Are you able to assess all statistics in the manuscript, including the appropriateness of statistical tests used?**

No, I do not feel adequately qualified to assess the statistics.

**Comments to author:**

In this study, Liu et al. have examined the role of the subunit of the SWI/SNF chromatin remodelling complex ARID1A, in the differentiation of human ESCs into neurons and cardiocytes. They show that deletion of ARID1A blocks cardiac differentiation and promotes spontaneous differentiation into neural stem cells and neurons, even in conditions that promote cardiocyte differentiation of wildtype cells. They then show that loss of Arid1a results in reduced chromatin opening and reduced transcription at cardiac genes while it results in increased transcription of neural genes irrespective of an effect on chromatin organisation. They also find that the repressor REST is significantly enriched at Arid1a bound locations in neural genes and that Arid1a and REST co-immunoprecipitate. They conclude that Arid1a regulates cardiac and neural development in hESCs by distinct mechanisms, the opening of chromatin at cardiac genes and the recruitment of REST at neural genes.

This is an interesting study that reports novel observations and will be of interest to a broad readership. However, while the first part of the study characterising the defects of hESCs with an Arid1a deletion (Figs 1-4) is well conducted and documented, the remaining part on the characterisation of the mechanisms of action of ARID1A (Fig. 5-7) is less convincing and does not fully support the conclusions of the authors. The authors will need to conduct new experiments to better support their conclusions or to seriously moderate their conclusions throughout the text including title, abstract, etc. My specific concerns are listed below.

1) The ATAC-Seq analysis shown in Fig. 5 is not convincing. Some of the changes in ATAC-Seq signals between WT and ARID1A-/- cells mentioned in the text are barely visible (e.g. in 5N, WT and KO profiles for EOMES, T, MESP1, HAND2 etc look very similar and only fractionally reduced in KO cells). I find it difficult to believe that such small differences are sufficient to explain the larger changes in gene expression observed at these loci. The authors show that binding sites for T and MEF2C are enriched at Arid1a sites and that the two factors co-immunoprecipitate with ARID1A. How do they exclude that loss of Arid1a results not only in (minor) changes in chromatin opening but also in a loss of T/MEF2C recruitment, and that the loss of transcriptional activity of these factors in turn results in the downregulation of target genes. Addressing this possibility would require to perform ChIP-Seq for T/MEF2C in wildtype and Arid1a-/- cells to examine recruitment of the two factors in the presence and absence of ARID1A.

2) Moreover, there is no data to support the authors' claim that T and MEF2C in turn recruit SWI-SNF complexes rather than acting directly to promote transcription. To demonstrate that regulation of cardiac genes relies on chromatin remodelling rather than transcriptional mechanism would require a more rigorous analysis, e.g. examining hESCs carrying point mutations in Smarca2/Smarc4 abolishing their ATPase activity. Without such experiments, the authors should tone down their conclusion that neurogenic and cardiac genes are regulated by separate mechanisms, transcriptional vs chromatin accessibility.

3) Fig. 7F, G: how were the 40 essential neurogenic genes and 40 essential cardiac genes selected. Were they selected in an unbiased manner or hand-picked to prove the point made by the authors?

4) Although the data in this study and previous analysis of mice carrying a Arid1aVG1068V mutation are compatible with a model of a direct binding of Arid1a to neuronal and cardiac genes, the experiments in this manuscript have not excluded that Arid1a is in fact recruited at least at some of its location by transcription factors. A demonstration that Arid1a binds DNA directly in hESCs, as claimed in p. 20, would require to engineer a mutation abolishing DNA-binding and demonstrate the loss of Arid1a recruitment. Here again the authors should tone down their conclusion.

5) The authors claim (e.g. in the abstract) that recruitment of REST by ARID1A is required to repress neurogenic genes. However this is not shown and the activity of REST is not examined in this study. A rigorous demonstration of this model would require, e.g. to engineer the recruitment of REST in Arid1a mutant cells at a derepressed neural gene and demonstrate that this is sufficient to rescue the ARID1A-/- phenotype and repress that gene. In the absence of such experiment, the authors should be more cautious and here again tone down their conclusion.

Minor points

Fig.1: to facilitate the reading, I suggest to mention briefly in the text the strategy to differentiate hESCs into multipotent cardiovascular progenitors and their progeny shown in 1A. Provide also some information on the generation of the interaction network in 1D.

Check the English as there are multiple instances of use of wrong words (e.g. "Controversy to previous report…" in p. 14; "..specifically modified to favorite human…" p. 19 and many others not listed here)

**Authors Response**

**Point-by-point responses to the reviewers’ comments:**

*Author responses: We deeply appreciate the constructive comments and suggestions from all three reviewers. We have revised the whole manuscript thoroughly according to the comments. We have reorganized and had native English speaker to revise the text of manuscript. Additionally, we further clarified experimental data/method description with more details and added more statistical analysis results into this resubmission according to the reviewer comments. All revisions are highlighted with yellow in the text.*

Reviewer comments:

Reviewer #1: This manuscript titled "Genome-wide studies reveal the essential and opposite roles of ARID1A in controlling human cardiogenesis and neurogenesis from pluripotent stem cells" by Liu et al., describes the role of chromatin remodeler protein ARID1A in regulating early human neural and cardiac differerntiation using a directed differentiation protocols. They use different techniques to interrogate the system and claim that ARID1A has two different roles in activating cardiogenesis in a chromatin remodeling dependent manner while repressing neurogenesis in a chromatin remodeling independent manner. They identify MEF2C and T as the transcription factors that drive initiation of cardiac mesoderm and cardiogenesis and show that ARID1A also interacts with neuronal repressor REST to prevent neurogenesis. This paper attempts to answer a very fundamental question in the developmental biology field as to how cells make different lineage decision but fails to answer it, largely as the authors start with a cell line that spontaneously differentiates from the beginning, indicating a fundamental defect in maintenance of pluripotency. The directed differentiation experiments fail to convince that ARID1A

Specific comments:

1. A very small percent of The ARID1A-/- cells spontaneously differentiate to neurons and express pan neuronal marker TUJ1 as well as more mature neuron marker MAP2 in ES cell culture conditions. The authors make the argument this is due to loss of ARID1A. Why only a small percentage of cells? If it is really ARID1A dependent shouldn't this be observed homogenously in most if not all of the cells? What prevents most other ARID1A-/- cells to not to undergo this kind of differentiation? Could the authors sub-clone their mutant colonies to have more homogenous population? The spontaneous loss of pluripotency towards a variety of cell types leads to a very different conclusion.

*Our answers: Thanks for asking the important question. This was raised by audience many times when we presented this work. Firstly, the simple answer is that the sporadic neural differentiation from ARID1A-/- hESCs is the true phenotype shown by the cells. RT-PCR, Western blot and scRNAseq (left figures) all confirmed that expression of ARID1A was fully depleted in ARID1A-/- hESCs. When both WT and ARID1A-/- hESCs were cultured under the exact same condition, we conclude whatever the phenotypic differences are due to loss-of-ARID1A.*

*Secondly, regarding the question “Why only a small percentage of cells? If it is really ARID1A dependent shouldn't this be observed homogenously in most if not all of the cells?”, our answer is that human ES cells are highly heterogeneous. Actually, the Figures 2E and 2F (left) from scRNAseq already distinguished 11 populations from undifferentiated hESCs according to the transcriptional heterogeneity. If hESCs are homogenous, theoretically, only two populations (undifferentiated and differentiated) could be identified here. The identification of 11 populations from hESCs indicated the high heterogeneity. Additionally, Nguyen et al. used single-cell RNA-seq of human induced pluripotent stem cells to reveal cellular heterogeneity and cell state transitions between subpopulations (Genome Res. 2018. 28: 1053-1066). They identified four subpopulations distinguishable on the basis of pluripotent state, including a core pluripotent population (48.3%), proliferative (47.8%), early primed for differentiation (2.8%), and late primed for differentiation (1.1%). They also quantified the transcriptional heterogeneity of subpopulations (please see left figure panel B). These results, together with our observation of sporadic neural differentiation in ARID1A-/- hESCs, suggest that loss-of-ARID1A could differentially impact on the differentiation potential of subpopulations. So that it is very possible a small subpopulation, such as subpopulation 4 of above figure, could rapidly differentiate into neural stem cells and neuron cells. However, we also detected enhanced expression of neural genes not only in cluster 10 but also in other ARID1A-/- hESCs compared with WT (left figure, Figure 2I), which suggests that these cells were under a bivalent condition and were not differentiated yet. This could also explain why ARID1A-/- hESCs gave rise to robust neural cells no matter under the cardiac or neural differentiation conditions, because loss-of-ARID1A presets the transcriptional program to neural fate. Once differentiation was triggered, the ARID1A-/- hESCs would tend to differentiate into neural cells.*

2.	The authors state in the abstract that "cardiac differentiation from ARID1A-/- hESCs was fully suppressed. ARID1A KO only decreased cardiac differentiation efficiency and didn't fully abolish it. In fact even in their directed differentiation system they achieve only 50% cardiomyocytes, and in the absence of ARID1A it goes down to 20%. This is hardly a cardiac to neural switch, especially as in WT differentiations the authors report a small percentage of neural cells. At best this indicates that ARID1A tilts the balance from mesoderm to ectoderm. Incidentally, the text describing Fig 3 is very difficult to follow and doesn't well represent the data.

*Our answers: Thanks for suggestions. We rewrote the abstract and toned down the text. In this study, our conclusion is that ARID1A could differentially control cardiac and neural differentiation from hESCs with distinct mechanisms. We don’t claim that ARID1A could fate switch mesoderm to ectoderm. For the Fig 3, we already reorganized the text and make it easier to follow and understand.*

3.	ARID1a loss in-vivo mice die E6.5 for lack of primitive streak formation, how the authors reconcile this? Does Over expression of ARID1A promote cardiac differentiation efficiency?

*Our answers: T is the key TF controlling mesoderm formation in primitive streak and expresses in the primitive streak during embryogenesis. We found loss-of-ARID1A reduced chromatin accessibility and expression level of T (left figures, Figure 5N-O). Loss-of-functional analysis is the classic strategy to confirm that a specific gene is required in organ development in vivo, we adopted the same strategy in human ES cell model to study ARID1A. Phenotypes from artificial gene overexpression could not conclude a gene is required for heart development. Although we appreciate the question of ARID1A overexpression, we feel all our dataset from knockout of ARID1A could be a stronger support for the conclusion.*

4.	Dissecting role of ARID1a affecting transcription by chromatin remodeling (cardiac) and chromatin remodeling independent (neural) is critical and could have given important insights into ARID1a role but is not addressed.

*Our answers: Our ATAC-seq, GO biological process analysis and qRT-PCR validation data in Figure 5B-F, 5G-J, 5K-O showed that chromatin accessibility on cardiac genes are dependent on ARID1A, whereas loss-of-ARID1A lead to both increased and non-changed chromatin accessibility on neurogenic genes. Additionally, qRT-PCR validated the expression changes of cardiac/neural marker genes in ARID1A vs. WT hESCs during differentiation. So we observed two different roles of ARID1A: (1) For cardiogenic genes, their chromatin accessibility was dependent on ARID1A. (2) For neurogenic genes, their transcription but not chromatin accessibility was directly affected by ARID1A.*

5.	Figure 1B and 1C needs more clarification. Are they GO biological process, molecular function or cellular components or anything else?

*Our answers: Those are GO biological process analyzed by Metacore software (Clarivate Analytics). We have provided more details in figure legends and text and results in resubmission. Thanks.*

6.	Figure 1E: Many subunits shown are not major SWI/SNF complex components. Authors need to change line 10 wordings. Showing all the major components of SWI/SNF and how their expression changes across these five different cell type will be useful to the readers.

*Our answers: Thanks. We revised this figure 1E and showed all key SWI/SNF complex components.*

7.	From Figure 1, it cannot be claimed that "ARID1A is involved in cardiac fate commitment from ESCs" as the authors do. It says at best that ARID1A expression is increased from hESCs to multipotent cardiovascular progenitor cells and cardiomyocytes and should be stated as such.

*Our answers: Thanks. We made changes in text and the Figure 1 title to “Loss-of-ARID1A induces spontaneous differentiation of hESCs”.*

8.	Figure 2D: Is it normal to have ~ 10% of WT cells express mature neuron marker MAP2? Does this means the culture condition employed here produces spontaneous differentiations of even WT cells?

*Our answers: Please refer to our answer to your first question regarding heterogeneity of stem cells. The figure from Genome Res. 2018. 28: 1053-1066 showed that MAP2 expression in subpopulation 4 of WT hiPSCs. And the data in Figure 2D were the mRNA expression which was detected by single cell RNA-seq. Although undifferentiated WT cells express MAP2, the MAP2 RNA and protein expression levels are very low (Figure 2K) compared with ARID1A-/-cells.*

9.	There are inconsistencies that the authors need to address between Figure 2D, and Figure 2K in MAP2 expression data from single cell RNASeq and FACS in WT and ARID1A-/- cells. They show different percentages.

*Our answers: Figure 2D shows the RNA expression level of MAP2 by scRNAseq, and Figure 2K shows the FACS results of MAP2 protein positive cells. It is reasonable that assays quantifying RNA and protein levels may not generate the exact same values. However, the trends of differences are same. Thanks for your question.*

10.	Supplementary Figure 2F: It appears that there is an increase of pre-cardiac and cardiac mesoderm markers EOMES and MESP1 in ARID1a-/- KO cells as well. Does this mean the KO cells are spontaneously differentiating to express these mesodermal markers, although to a lower extent compared to the neural markers?

*Our answers: Thanks for the important question. Please refer to our answer to your first question regarding heterogeneity of stem cells. The figure from Genome Res. 2018. 28: 1053-1066 showed that expression of mesoderm markers in some subpopulations of WT hiPSCs. In this resubmission, we provide statistical results of Figure S2f. P values between KO and WT cells for each gene were not significant. We also included new RT-qPCR data to show that there were also not significant between undifferentiated WT and KO cells for each gene (new supplementary figure 2H).*

11.	Figure 3L: Why do WT cells shows mature neurons in a cardiac differentiation system? What is the cardiac differentiation efficiency? The authors need to explains this? Also they need to discuss the lack of endothelial cells and fibroblast in ARID1A-/- cells and need to comment if ARID1A facilitates differentiation of endothelial and fibroblasts cells.

*Our answers: The percentage of WT cells expressing mature neuron markers was low and the expression level of mature neuron markers were also very low. And please refer to the last paragraph of our answer to your first question regarding heterogeneity of stem cells. The cardiac differentiation efficiency was already shown in Figure 4B. We provided the potential explanation in the page 16 of text. Both cardiac fibroblasts and endothelial cells are derived from mesoderm. Since loss-of-ARID1A affects expression of mesoderm marker T and decreased cardiac differentiation, it is reasonable that differentiation of fibroblasts and ECs from ARID1A-/- cells were reduced. We added a sentence in the text of page 9-10.*

12.	Figure 5: It would be useful to perform ATAC-seq on the hESCs as spontaneous neural differentiation is observed in these cells. This would also add insights if ARID1A acts in the ES cell stage to alter the chromatin landscape of the cell in order to facilitate lineage specific gene expression programs.

*Our answers: Thanks for the suggestions. Although ATAC-seq on hESCs could add insights, the scRNAseq on hESCs already revealed the gene expression landscape and found the globally increased expression of neurogenic genes in ARID1A null hESCs. Expression of cardiac genes were not increased in ARID1A null hESCs (Supl Figs. 2F-H). Additionally, our ATAC-seq in day 4 hESCs showed that chromatin accessibility on neurogenic genes were not uniformly altered upon ARID1A knockout. Therefore, we don’t expect uniform chromatin change would occur on neural genes in undifferentiated ARID1A null hESCs.*

13.	Figure 5E: y-axis scale would be necessary as it is difficult to see if KO has high chromatin accessibility than WT.

*Our answers: In this resubmission, we labeled the y-axis in Figure 5E.*

14.	Figure 5E and F: While authors claim that there is increase in chromatin accessibility at the promoters of ZIC1, LHX5, FABP7 and MAP2, in reality the increase is very minor and WT tracks show almost similar chromatin accessibility. How similar chromatin accessibility gives rise to such significant differences in gene expression (Figure 5F) is not at all clear and needs more explanation. With the same logic, the repression of cardiac genes in ARID1A-/- mutants (Figure 5 O)could not be explained based on the chromatin accessibility patterns. Moreover, for Mesp1 and T the decreased chromatin accessibility does not result in change in gene expression. Thus it is difficult to understand if the changes in ARID1A mediated chromatin accessibility cause the changes in gene expression.

*Our answers: We feel sorry for the under-zoomed data from Genome Browser in Figure 5 of the previous submission. We were trying to include the promoter and coding regions as much as possible for each example gene, which affected the view of target genomic regions showing alerted chromatin accessibility. In this resubmission, we zoomed in the promoter regions for each example and framed out the regions showing alerted chromatin accessibility in Figure 5E and 5N. We also added two more neural genes showing prominently changed chromatin accessibility in the Figure 5E.*

15. Figure 6: These ARID1A ChIPs were carried out in hESCs and shows that ARID1A occupies many neural genes promoters as well as cardiac promoters. Why does ARID1A occupy only cardiac and neural promoters at this stage? Is it guarding the fate of these cells only to these two differentiation lineages? How many total ARID1A peaks detected? How many of them occupy neural genes and how many of them occupy cardiac genes? Is it an independent function of ARID1A or of BAF complexes in general? This should be compared to known data. Most importantly what about all other processes that happens in hESCs, or does ARID1A function specifically and only in cardiac and neural processes? These questions needs to be addressed.

*Our answers: Thanks for the key question. As mentioned in the introduction section, previous in vivo data found that loss-of-functional Arid1a led to defective heart and brain formation. This is why we aimed to study the role of ARID1A under heart and neural development from human ESCs. And our experimental data showed that during differentiation ARID1A deletion could not affect endodermal markers expression (Supplementary Figure 7C), which also indicated that ARID1A might specifically affect cardiac and neural lineages differentiation. Regarding whether ARID1A regulates other process, it is possible but out of the scope of this study.*

*After we observed KO of ARID1A significantly affected cardiac and neural differentiation from hESCs, we performed ATAC-seq, ChIP-seq and other analyses to address the mechanisms by ARID1A underlying those two processes. ChIP-seq found ARID1A occupies 6419 genes in hESCs (Figure 7C), of which 514 are ESSENTIAL neurogenic genes and 261 are ESSENTIAL cardiogenic gene (Figures 7D, E), which are the most important genes to control cardiogenesis and neurogenesis. We never claimed ARID1A only occupied cardiac and neural genes in the manuscript. We only want to provide solid evidences to address why loss-of-ARID1A affect cardiac and neural development.*

*Since ARID1A is a DNA-binding component of SWI/SNF complex, and previous studies found that loss-of-ARID1A reduced the chromatin accessibility of SWI/SNF. So, we conducted ATAC-seq to characterize the altered chromatin accessibility on cardiac and neurogenic genes. We found loss-of-ARID1A led to globally and uniformly reduced chromatin accessibility on cardiac genes, but increased and non-changed chromatin accessibility on neural genes. These could allow us to conclude chromatin accessibility on cardiac genes is ARID1A dependent, whereas chromatin accessibility on neural genes is not ARID1A dependent. Furthermore, we found neurogenic transcription is globally increased in ARID1A-/- cells and ARID1A could associate with a transcriptional repressor, REST. REST is known to repress transcription of neural genes during mouse neurogenesis. So that these results suggest that ARID1A could very likely repress transcription of neural genes via binding REST.*

*Again, we just aimed to uncover the different mechanisms by ARID1A under heart and neural development from human ESCs. The roles ARID1A and SWI/SNF in many other processes are not the focus of this study.*

Minor:

1. line 5 : neural crescent cells should be neural crest cells

2. line 51 : Ariad1a typo needs to be fixed

*Our answers: We apologize for the typos. We had a native English speaker to revise the text of resubmission.*

Reviewer #2: Liu et al. hypothesize that distinct ARID1A-dependent chromatin remodeling mechanisms govern cardiogenesis and neurogenesis. ARID1A has known roles in cardiac and neural development in vivo. The manuscript utilizes hESC differentiation and single-cell sequencing approaches to address the mechanism of ARID1A-SWI/SNF chromatin remodeling in cardiac versus neural lineage differentiation. Comparisons with ARID1A binding (via ChIPseq) and ARID1A-dependent chromatin accessibility changes (via ATACseq) are provided. This is manuscript is

suitable for Genome Biology readership. However, it's very rough around the edges and the data presented lack statistical rigor. As a result, many of the conclusions raised are difficult to interpret. This makes it hard to both properly evaluate and judge the novelty of the findings in the manuscript. Specific comments are listed below.

Comments:

* Please provide a reviewer token to the GEO submission. Please include a supplemental table with descriptions of the samples submitted and number of replicates performed.

*Our answers: We feel sorry for missing the token information for GSE139260. Here it is: azstasmudhitpyz. The sample information was listed in Supplemental Sample Information.*

* Figure panels should be introduced alphabetically in the text.

*Our answers: Thanks for reviewer’s correction. We made changes accordingly.*

* Genomic statements are frequently made with only visual verification from plots. Increase use of statistical tests to backup genomic findings throughout manuscript.

*Our answers: Thanks for reviewer’s insightful suggestion. Indeed, we did statistical tests on all comparison, including scRNA-seq, ATAC-seq, and ChIP-seq data. We re-wrote the statistics part in the Method and added p-values in the text, corresponding figures and figure legends.*

* Detailed descriptions of the methodology and data analysis are required. Please provide descriptions of the statistical tests used.

*Our answers: Thanks. More details in the Method section have been added.*

* Please make sure all of the text in figures are legible, or remove if unnecessary. Plot text is frequently too small to read. Several figure panels show text that impossible to read.

*Our answers: We feel sorry for the problems. We have thoroughly revised the font size in all figures and made it as large as possible.*

* Frequent grammatical, syntax and spelling errors are found throughout the manuscript and should be corrected. Please have a professional editor review the manuscript.

*Our answers: We apologize for the typos. We used automatic grammar check and invited a native English writer to revise the text of resubmission.*

* Refer to SWI/SNF as SWI/SNF chromatin remodeling complex rather than epigenetic complex. Our answers: Thanks for suggestion. We made changes accordingly in the text.

* Student's t-test should only be used with normally distributed data with similar variance between groups. Consider using a nonparametric test for certain analyses where high sampling variance is observed.

*Our answers: Thanks for suggestion. We have explained specific statistical test methods used for different types of data analyses, e.g. for flow cytometry and qPCR data, parametric t-test was used for normally distributed data with similar variance between two groups, and a nonparametric t-test was used for certain analyses with high sampling variance. A nonparametric Wilcoxon test for scRNA-seq gene expression difference, and the Fisher’s text for cell numbers difference obtained by scRNA-seq, respectively. More details can be found in the Methods section as well as figure legends for corresponding panels.*

* In the abstract, it is stated that, 'ARID1A recruited T and MEF2C to cadiogenic genes,' but there's no data supporting these conclusions. Either remove these concluding statements or include T or MEF2C ChIPseq or ChIPseq data comparisons.

*Our answers: Thanks for suggestion. We have removed this statement from the revised version and toned down our conclusions.*

* It is not stated how ATAC-seq and ChIP-seq peaks were functionally linked to genes. It is assumed this is based on proximity to gene, but it is not clear whether this is within a certain distance to gene TSS or over gene bodies, etc. This information is not found within the manuscript and should be included.

*Our answers: Sorry for the confusion. We have clarified the way we linked genomic regions to specific genes. As we explained in the Methods, “The open chromatin regions detected by ATAC-seq or ARID1A binding sites identified by ARD1A ChIP-seq were linked to specific genes, if they locate either within upstream 10kb from TSS, or 5’UTR, or exon/intron (gene body) of the genes.”*

* In the results section, 'Therefore, scRNA-seq results demonstrate that loss-of-ARID1A in hESCs exclusively increases transcription of neural-associated marker genes and induces spontaneous neural differentiation, indicating ARID1A plays an important role in repressing neural commitment from hESCs' is a bold statement that is only backed by a few representative cell-marker genes. A more quantitative analysis should be performed to support this claim, such as enrichment tests for cell type gene signatures in all clusters, then can compare observed/expected gene set enrichment ratios. Or, compare relative mean expression values for all genes in a cell type signature between clusters, etc.

*Our answers: Our conclusion was made based on results from scRNAseq data from Figure 2 and Figure 3, which included multiple neural genes. Additionally, more neurogenic genes were included in supplementary Figure 4E. Enrichment tests for cell type gene signatures within clusters were shown in supplementary Figure 5. Furthermore, average signature gene expression of clusters was shown in supplementary Figure 4F-K.*

* Number of ATAC-seq replicates for each condition are not stated. Since edgeR was used to calculate differential accessibility, at least 2 replicates are inferred, but this n should be specified. Also, please state what normalized values were used for differential accessibility analysis. Further, 'We then constructed a final set of unique regions by merging overlapped open chromatin regions recognized from different samples/conditions' is vague and should be more specific. Were MACS2 peaks firstly intersected between biological replicates, then taken the union between conditions? "Merged overlapping open chromatin regions" are assumed to be the input for edgeR analysis, but please confirm this in the methods section.

*Our answers: We apologize that we didn’t make this clear in the original manuscript. We had two replicates for ATAC-seq, both WT and ARID1A-/-. As we emphasized in the Methods section, “Open chromatin regions for each sample were identified first from peak calling of individual ATAC-seq using MACS2 [61] with broad peak option and cutoff of q < 0.1. We then constructed a final set of unique regions for differential analysis by merging overlapped open chromatin regions recognized from different samples/conditions. The read abundance of each open region was counted for each individual sample, respectively, by using pyDNase [62]. A software, edgeR [63, 64], was employed to perform differential accessibility analysis on the read counts of each chromatin region for WT and ARID1A-/- samples. Differentially accessible open chromatin regions between WT and ARID1A-/- were determined by specific cutoffs, FDR-adjusted p-value less than 0.01 and amplitude of fold change (in log scale with base 2) larger than 0.5.”*

* Fig. 1E - legend states 'Genes expression profiles of SWI/SNF subunits in different cardiovascular lineages by whole transcriptome sequencing.' Then proceeds to show expression data of ARID1A, ARID1B, and 7 genes encoding non-SWI/SNF ARID domain containing proteins, and SMARCA2 / SMARCC2. The non-SWI/SNF ARID subunits should be omitted from this analysis since, and potentially include other SWI/SNF subunits, like SMARCA4.

*Our answers: We revised to show all essential SWI/SNF components in this resubmission.*

* Fig. 1F - RT-PCR analysis shows only a modest, 1 log2 fold increase in mRNA expression for ARID1A during differentiation. This modest increase may not result in altered protein expression and should be validated by a protein-based assay such as WB.

*Our answers: Thanks for suggestion. We repeat the differentiation experiment, followed with q-RT-PCR again. We also added expression dynamic of T and MEF2C, which showed consistent expression dynamic as in Western blot of Figure 7H.*

* Fig. 1K - the difference between red and white arrows, if any, is not clear. Choose one color if no distinction.

*Our answers: The two colors indicate two typic types of morphologically different cells.*

* Fig. 2B-C - Need to apply a statistical test to quantitatively support these claims, such as wilcox test.

*Our answers: As reviewer suggested, we provided p-values from Wilcox test for the genes in the figures. It is not surprised to see highly significance (maximum p-value < 2.4E-13) for such gene expression comparisons between WT and ARID1A-/- based on scRNA-seq data.*

* Fig. 2D - Again, a statistical test of proportions like Fisher's can better support this claim

*Our answers: We calculated p-values based on Fisher’s exact test. All had very significant differences with regard to percentages of marker gene-expressed cells (maximum p < 1.1E-14).*

* Fig. 2I - while this is a nice visual, it is redundant with Fig. 2G and should be moved to supplemental

*Our answers: Fig. 2I and Fig. 2G are somehow different. Fig. 2I shows the distribution of cells with expressed gene makers, which underlines the difference between WT and ARID1A-/-. However, Fig. 2G represents gene expression in each cluster and emphasizes the difference between cluster 10 and the others.*

* Supp. Fig. 2D - Text states " Loss-of-ARID1A did not affect the expression of pluripotency... genes." but POUF51 and SOX2 appear shifted in expression, potentially to a statistically significant degree. Please include summary statistics for any plots shown like this.

*Our answers: Thanks. Indeed, OCT-4 underwent a notable change of gene expression after KO (p = 5.3E-57), while SOX2 didn’t have a significant alternation.*

* Fig. 3C-D - same comments as Fig. 2B-C

*Our answers: As we pointed out in Fig. 2B-C, all selected genes in the figure had significant gene expression change, either up-regulated (maximum p < 8.3E-100) or down-regulated (p = 0).*

* Fig. 3I - same comments as Fig. 2I

*Our answers: Please see our response to Fig. 3I.*

* Supplementary Fig. 4 panels G-K are not referenced in the text.

*Our answers: Sorry for the misleading information. Please read revised page 9 text “Average gene expression in each cell cluster (ARID1A-/- vs. WT) (Supplementary Figs. 4F-K) was consistent with the results of violin plots”.*

* Fig. 4G-J - the difference between panels G and H is not clear. Likewise, for panels I and J.

*Our answers: Figure 4G-H show the IHC results of TUJ1/MAP2 and CTNT in WT and ARID1A null cells. The Figure 4I-J indicate the IHC results of SOX1 and SOX2 staining in WT and ARID1A null cells.*

* Supp. Fig. 6G and 6K are unnecessary; constructs should be omitted and referenced instead.

*Our answers: Those were removed.*

* Fig. 5E - genomic tracks of ATAC-seq data should have y-axis and data type specified in legend.

There is currently no information provided. Are these bedgraphs of normalized ATAC signal per bp? MACS2 enrichment score? etc. The ChIP-seq tracks e.g. in Fig. 6F have a y-axis, so something similar should be provided for these ATAC-seq tracks.

*Our answers: Sorry for the confusion. We added the number in the y-axis of figures for ATAC-seq genomic track. The number represents were explained in the Methods, “The UCSC genome browser custom track was made to visualize differences of chromatin accessibility between WT and ARID1A-/- for selected regions. The read numbers were normalized by total numbers of high quality reads in each sample, then multiplied by ten millions. The normalized averaged counts for replicates under the same condition were presented in the figures.”*

* Fig. 6F, 6I - 'Example views of ARID1A occupancies...' should be more clear. Like in Fig. 5E, please state what data are being shown. Reads per million per base? etc.

*Our answers: As added in page 27 Method section, the number on y-axis represents sequence counts normalized by total numbers of high quality reads in each sample, then multiplied by ten millions.*

* In Fig. 7I-K and Suppl. Fig. 7, the Co-IP data do not include proper Input controls. The input fractions should be probed the antibodies used to detect interacting proteins.

*Our answers: For Figure 7I-K and Supl Figure 7, the Figure 7H could serve as input control for REST, T, MEF2C.*

* While the FIG. 7 ARID1A vs. REST ChIP-seq comparisons provide support for the model, there's no comparisons between ARID1A and T or MEF2C.

*Our answers: Thanks. A major conclusion of this study is that ARID1A globally controls chromatin accessibility on essential cardiogenic genes. And we have provided sufficient evidences. We also toned down the description of ARID1A and T/MEF2C interaction in text. Full characterization of the role of TFs, including T/MEF2C, in guiding SWI deposition will be our next study.*

Reviewer #3: In this study, Liu et al. have examined the role of the subunit of the SWI/SNF chromatin remodeling complex ARID1A, in the differentiation of human ESCs into neurons and cardiomyocytes. They show that deletion of ARID1A blocks cardiac differentiation and promotes spontaneous differentiation into neural stem cells and neurons, even in conditions that promote cardiomyocyte differentiation of wildtype cells. They then show that loss of Arid1a results in reduced chromatin opening and reduced transcription at cardiac genes while it results in increased transcription of neural genes irrespective of an effect on chromatin organization. They also find that the repressor REST is significantly enriched at Arid1a bound locations in neural genes and that Arid1a and REST co-immunoprecipitation. They conclude that Arid1a regulates cardiac and neural development in hESCs by distinct mechanisms, the opening of chromatin at cardiac genes and the recruitment of REST at neural genes.

This is an interesting study that reports novel observations and will be of interest to a broad readership. However, while the first part of the study characterizing the defects of hESCs with an Arid1a deletion (Figs 1-4) is well conducted and documented, the remaining part on the characterization of the mechanisms of action of ARID1A (Fig. 5-7) is less convincing and does not fully support the conclusions of the authors. The authors will need to conduct new experiments to better support their conclusions or to seriously moderate their conclusions throughout the text including title, abstract, etc. My specific concerns are listed below.

*Our answers: Thank you very much for the suggestions. The abstract has been rewritten and we moderated the conclusions as suggested, “Global chromatin accessibility on cardiogenic genes is dependent on ARID1A, whereas transcriptional activity of neurogenic genes is under control by ARID1A, possibly through ARID1A-REST/NRSF interaction.”.*

1) The ATAC-Seq analysis shown in Fig. 5 is not convincing. Some of the changes in ATAC-Seq signals between WT and ARID1A-/- cells mentioned in the text are barely visible (e.g. in 5N, WT and KO profiles for EOMES, T, MESP1, HAND2 etc look very similar and only fractionally reduced in KO cells). I find it difficult to believe that such small differences are sufficient to explain the larger changes in gene expression observed at these loci. The authors show that binding sites for T and MEF2C are enriched at Arid1a sites and that the two factors co-immunoprecipitate with ARID1A. How do they exclude that loss of Arid1a results not only in (minor) changes in chromatin opening but also in a loss of T/MEF2C recruitment, and that the loss of transcriptional activity of these factors in turn results in the downregulation of target genes. Addressing this possibility would require to perform ChIP-Seq for T/MEF2C in wildtype and Arid1a-/- cells to examine recruitment of the two factors in the presence and absence of ARID1A.

*Our answers: We feel very sorry for the under-zoomed data from Genome Browser in Figure 5 of the previous submission. We were trying to include the promoter and coding regions as much as possible for each example gene, which affected the view of target genomic regions showing alerted chromatin accessibility. Indeed, we selected the genes presented in the figures based on the statistical analysis with specific cutoffs (see Methods) and genes’ functions. In this resubmission, we zoomed in the promoter regions for each example and framed out the regions showing significantly alerted chromatin accessibility in Figure 5E and 5N.*

*Thank you for raising the question “ How do they exclude that loss of Arid1a results not only in (minor) changes in chromatin opening but also in a loss of T/MEF2C recruitment, and that the loss of transcriptional activity of these factors in turn results in the downregulation of target genes.” This is actually the most frequently asked question when we present this study in public. Below is our answer.*

*During cardiogenesis, T and MEF2C are not expressed in undifferentiated hESCs (Figure 7H, left). In this study, we performed ARID1A ChIP-seq in undifferentiated hESCs (see Figure 6 data) and found it occupied many essential cardiogenic genes including mesodermal TF, T and cardiogenic TFs, NKX2.5, ISL1, GTA4/5, TBX3/5 etc., as well as cardiac function related genes such as CTNT, MYH7. This result, together with the significantly decreased chromatin accessibilities on those cardiogenic genes in ARID1A-/- cells (Figure 5 ATAC-seq results), strongly suggest that ARID1A globally pre-determines the chromatin accessibility on cardiogenic genes ever since the pluripotent stem cell stage, when T and MEF2C are not yet expressed. Accordingly, our conclusion is that ARID1A predominantly determines chromatin accessibility on cardiogenic genes before transcription of cardiogenic genes. We do agree with the reviewer that T/MEF2C could possibility associate with ARID1A to target on some other cardiogenic genes at the relatively later stages of cardiogenesis, and we added a paragraph in the page 15 of Discuss section to echo with reviewer 1. However, functional study of such later stage T/MEF2C-ARID1A association could be our next study.*

2)	Moreover, there is no data to support the authors' claim that T and MEF2C in turn recruit SWI-SNF complexes rather than acting directly to promote transcription. To demonstrate that regulation of cardiac genes relies on chromatin remodeling rather than transcriptional mechanism would require a more rigorous analysis, e.g. examining hESCs carrying point mutations in Smarca2/Smarc4 abolishing their ATPase activity. Without such experiments, the authors should tone down their conclusion that neurogenic and cardiac genes are regulated by separate mechanisms, transcriptional vs chromatin accessibility.

*Our answers: Thanks for the suggestions. We have revised the manuscript and toned down that conclusion.*

3)	Fig. 7F, G: how were the 40 essential neurogenic genes and 40 essential cardiac genes selected. Were they selected in an unbiased manner or hand-picked to prove the point made by the authors?

*Our answers: The essential neural and cardiac gene lists are from an unbiased database, the Human Protein Atlas database (https://www.proteinatlas.org/). In Figure 7D and E (left), we showed that REST tend to co-occupy with ARID1A on neural genes than cardiac genes (312 vs. 47). In Figure 7F, G, we showed 40 representative genes according to the order of*

*their differences of chromatin accessibility (DCA).*

4)	Although the data in this study and previous analysis of mice carrying a Arid1aVG1068V mutation are compatible with a model of a direct binding of Arid1a to neuronal and cardiac genes, the experiments in this manuscript have not excluded that Arid1a is in fact recruited at least at some of its location by transcription factors. A demonstration that Arid1a binds DNA directly in hESCs, as claimed in p. 20, would require to engineer a mutation abolishing DNA-binding and demonstrate the loss of Arid1a recruitment. Here again the authors should tone down their conclusion.

*Our answers: Previous studies have proved the DNA-binding capacity of ARID1A. Thus, we cited finding from other labs to provide rational for performing ChIP-seq of ARID1A. In the current resubmission, we have modified and toned down the discussion section in page 17 of text, as suggested. Thanks.*

5)	The authors claim (e.g. in the abstract) that recruitment of REST by ARID1A is required to repress neurogenic genes. However, this is not shown and the activity of REST is not examined in this study. A rigorous demonstration of this model would require, e.g. to engineer the recruitment of REST in Arid1a mutant cells at a depressed neural gene and demonstrate that this is sufficient to rescue the ARID1A-/- phenotype and repress that gene. In the absence of such experiment, the authors should be more cautious and here again tone down their conclusion.

*Our answers: Thanks for the suggestions. In ARID1A-/- hiPSCs, when ARID1A is totally depleted, REST could not be engineered to target the specific neurogenic genes that it occupied in WT hiPSCs. Technically, such experiment is not doable. So that we rewrote and toned down abstract and page 17 of Discussion section as suggested.*

Minor points

Fig.1: to facilitate the reading, I suggest to mention briefly in the text the strategy to differentiate hESCs into multipotent cardiovascular progenitors and their progeny shown in 1A. Provide also some information on the generation of the interaction network in 1D.

*Our answers: We have added some detailed description of these results into the current text page 5.*

Check the English as there are multiple instances of use of wrong words (e.g. "Controversy to previous report..." in p. 14; "..specifically modified to favorite human..." p. 19 and many others not listed here)

*Our answers: We apologize for the typos. We used automatic grammar check and invited a native English speaker to revise the text of resubmission.*

**Second round of review**

**Reviewer 1**

The authors have revised their manuscript and attempted to address our concerns. In the cover letter the authors state "it seems that reviewer 2 lacks sufficient background knowledge in heterogeneity of human stem cells and developmental processes. Many of his/her concerns have already been addressed by others or in our manuscript, but our detailed data were somehow ignored." I assure the authors and the editor that my lab has over 15 years of experience differentiating mouse and human ES and iPS cells, and we are keenly aware of heterogeneity and developmental processes. The supposed marked heterogeneity in hESCs is not statistically supported as it is easy to overcluster scRNAseq data, which is the case in the data presented. There are no other reports of such intrinsic heterogeneity. Regardless, the point made by the authors indicate then that loss of ARID1A would simply bias one population's spontaneous differentiation over the other. Our surprise over the 50% cardiac in the presence of 10% neural is based on our considerable experience in differentiation of human iPS cells, where we routinely, with very different protocols, achieve 70-90% cardiac cells with 0% neural or any other neuroectoderm differentiation. Therefore the authors' protocols are not sufficiently rigorous to drive specific lineages, and therefore the shift from one to the other in terms of proportion is clear, but not indicative of a fate switch. Again regardless of this, the authors have toned down their conclusions, but this diminishes the impact of the work, and their proposed mechanisms are not consistent with the observations.

Other aspects of the work are improved, and the ATACseq figures are more convincing, although that these specific accessible regions are important for expression of the nearby genes is not clear, especially if the promoters are unaffected. The connection to T and MEF2C is still weak, especially if the actual result is a skewed differentiation from pluripotency.

Finally the authors' results should be contrasted with other publications that arrive at somewhat different conclusions regarding the mechanism by which loss of ARID1A impairs cardiogenesis (https://www.ncbi.nlm.nih.gov/pubmed/26070559, https://www.ncbi.nlm.nih.gov/pubmed/32039194 )

**Reviewer 2**

During the last submission, much the criticism centered on lack statistical rigor, poor data presentation and lack of experimental details. Liu et al. do a good job responding to the reviewer's comments and the paper has improved, but it is still not ready for publication in Genome Biology.

*	Based on GSE139260, it appears that only one ARID1A ChIP replicate is provided (n=1). Was only one ARID1A ChIP and one input replicate experimentally generated and sequenced? At least 2 independent biological ChIP replicates should be performed, analyzed and reported in the findings of the paper. Please make sure you include a supplemental table of all of the the individual samples submitted to GEO (scRNAseq, ATAC-seq and ChIP-seq).

*	Only ARID1A ChIPseq samples are included in GEO GSE139260 submission token. Please include the other genomic datasets (scRNAseq and ATACseq) with this submission. All of the datasets should be deposited in GEO with linked accession numbers and file sets.

*	The first sentence of the results section still refers to the "SWI/SNF epigenetic complex." As previously mentioned, please instead refer to it as the SWI/SNF chromatin remodeling complex.

*	As mentioned in the previous review, in Fig. 7h, the input fractions should be probed with the same antibodies used to detect interacting proteins from the pulldowns. The use of GAPDH is not appropriate.

*	Text legibility has improved for all the figures. However, axes are still not labeled or suitably described in the legend. E.g. Fig. 3c,d,j,k,i. Fig. 3c-d legend states "Violin plots showing expression of..." but does not indicate what these values are. Please be more transparent in the presented values. Are these normalized counts from Seurat? Log or linear? This information is critical to interpretation of the data presented.

*	Certain statistics are unclear. For example, Fig. 3e-f legend states "* p < 3.13E-163 (Fisher's exact test) (e)", but 6 tests are made. Does that indicate p < 3.13E-163 is the least significant (highest) probability from all 6 tests? Please clarify these points and provide a better description the of tests being performed.

*	Y-axis scales for genome browser tracks were clarified in the methods section, but these should be added to the respective legends and/or plots for increased legibility while reading. E.g. "Signal is displayed as reads per bp per 10 million reads (RPB10M)."

**Reviewer 3**

The authors have addressed most of my concerns and those of the other referees in their response letter and in the revised manuscript. The manuscript has improved somewhat as a result. The experimental evidence for the main conclusions of the study remains somewhat shallow. In fairness it wasn't feasible during a short revision period to obtain additional experimental support for the conclusions as suggested in the first review, and the authors have to some extent toned down their conclusions in the revised version, as requested.

The quality of the English remains low and the manuscript is still difficult to read. Some of the sentences added to the revised manuscript are hard to understand (example p. 9: "Feature plots from integrative analysis further distinguished distribution patterning of cells, which expressed signature neural and cardiac genes, across all clusters"). The manuscript will need thorough editing before publication.

**Authors Response**

**Point-by-point responses to the reviewers’ comments:**

Below are our responses to the 2nd-round reviewer comments:

Reviewer reports:

Reviewer #1: The authors have revised their manuscript and attempted to address our concerns. In the cover letter the authors state "it seems that reviewer 2 lacks sufficient background knowledge in heterogeneity of human stem cells and developmental processes. Many of his/her concerns have already been addressed by others or in our manuscript, but our detailed data were somehow ignored." I assure the authors and the editor that my lab has over 15 years of experience differentiating mouse and human ES and iPS cells, and we are keenly aware of heterogeneity and developmental processes. The supposed marked heterogeneity in hESCs is not statistically supported as it is easy to overcluster scRNAseq data, which is the case in the data presented. There are no other reports of such intrinsic heterogeneity.

*Answer: We can’t agree that “There are no other reports of such intrinsic heterogeneity”. In our first response to the reviewer (reviewer #2 in round one), we provided a recent paper by Nguyen et al. who used single-cell RNA-seq of human induced pluripotent stem cells to reveal cellular heterogeneity and cell state transitions between subpopulations (Genome Res. 2018. 28: 1053-1066). Additionally, PubMed showed 596 papers cover this topic, e.g.*

*J Clin Invest. 2011. 121(3):1217-1221*

*Stem Cells 2012. 11:2423-36*

*Nature Reviews Molecular Cell Biology 2013.14: 357–368*

*Genome Res. 2018. 28: 1053-1066*

*In this study, we have already got full sets of scRNAseq and lots of experimental validation data to support our conclusion. If it were over-clustered by scRNAseq, FACS and qRT-PCR would not be able to validate the results.*

Regardless, the point made by the authors indicate then that loss of ARID1A would simply bias one population's spontaneous differentiation over the other. Our surprise over the 50% cardiac in the presence of 10% neural is based on our considerable experience in differentiation of human iPS cells, where we routinely, with very different protocols, achieve 70-90% cardiac cells with 0% neural or any other neuroectoderm differentiation. Therefore the authors' protocols are not sufficiently rigorous to drive specific lineages, and therefore the shift from one to the other in terms of proportion is clear, but not indicative of a fate switch. Again regardless of this, the authors have toned down their conclusions, but this diminishes the impact of the work, and their proposed mechanisms are not consistent with the observations.

*Answer: The differentiation method led by Dr. Lei Yang (contact author of the paper) was first published in Nature 453 (7194), 524-528 with over 1400 citations (see screenshot from the google scholar). We do not know what kind of special method this reviewer referred to and uses in his/her own lab. We would appreciate if the reviewer could specify this in the comments. However, if the method only generates heart cells, it might not be suitable for modeling human embryogenesis. Because embryos make not only cardiac, but also neural, liver, pancreatic, blood cells etc.*

Other aspects of the work are improved, and the ATACseq figures are more convincing, although that these specific accessible regions are important for expression of the nearby genes is not clear, especially if the promoters are unaffected. The connection to T and MEF2C is still weak, especially if the actual result is a skewed differentiation from pluripotency.

*Answer: Strong connections were presented in the manuscript between genes and chromatin accessible regions on the gene promoters. We showed changes of ATACseq signals in the promoter regions of genes. Expression levels of these genes were quantified by qRT-PCR as well.*

Finally the authors' results should be contrasted with other publications that arrive at somewhat different conclusions regarding the mechanism by which loss of ARID1A impairs cardiogenesis (https://www.ncbi.nlm.nih.gov/pubmed/26070559,https://www.ncbi.nlm.nih.gov/pubmed/32039194 )

*Answer: Those two studies the reviewer mentioned here do not have conflict conclusions with ours. They, however, just revealed different mechanistic conclusion with focus on one or two interacting genes. Those two studies neither had comprehensive genome-wide analyses, nor investigated both cardiogensis and neurogenesis in one study.*

Reviewer #2: During the last submission, much the criticism centered on lack statistical rigor, poor data presentation and lack of experimental details. Liu et al. do a good job responding to the reviewer's comments and the paper has improved, but it is still not ready for publication in Genome Biology.

* Based on GSE139260, it appears that only one ARID1A ChIP replicate is provided (n=1). Was only one ARID1A ChIP and one input replicate experimentally generated and sequenced? At least 2 independent biological ChIP replicates should be performed, analyzed and reported in the findings of the paper. Please make sure you include a supplemental table of all of the the individual samples submitted to GEO (scRNAseq, ATAC-seq and ChIP-seq).

*Answer: Many papers published in high-impact journals had no replicates for ChIP-seq. Here are some examples:*

*(a) The histone mark H3K36me2 recruits DNMT3A and shapes the intergenic DNA methylation landscape (Nature 573, 281–286, 2019).*

*(b) Mouse models of neutropenia reveal progenitor-stage-specific defects (Nature 582, 109–114, 2020).*

*(c) Coordinated alterations in RNA splicing and epigenetic regulation drive leukaemogenesis (Nature 574, 273–277, 2019).*

*(d) Pervasive Chromatin-RNA Binding Protein Interactions Enable RNA-Based Regulation of Transcription (Cell 178 (1), 107-121, 2019).*

*(e) A Multiscale Map of the Stem Cell State in Pancreatic Adenocarcinoma (Cell 177 (3), 572-586, 2019)*

*(f) A Dominant-Negative Effect Drives Selection of TP53 Missense Mutations in Myeloid Malignancies (Science 365 (6453), 599-604, 2019)*

*(g) Therapeutic targeting of preleukemia cells in a mouse model of NPM1 mutant acute myeloid leukemia (Science 31 Jan 2020)*

*Of course, we totally understand the reason why the reviewer emphasized the importance of replicates in terms of statistical significance. Unfortunately, we can’t generate more ChIP-seq data during such a short revision period, particularly our school has been locked down due to COVID-19. But the good news is that we previously used another antibody from rabbit to perform ARD1A ChIP-seq in the same biological system as shown in the current manuscript. Actually, in the paper “Distinct structural classes of activating FOXA1 alterations in advanced prostate cancer” published in Nature (571, 413–418) in 2019, the authors used different antibodies as replicates.*

*Below, we would like to show the similarity between two ARID1A ChIP-seq data achieved from different antibodies.*

*A) Venn diagram shows that about 72% of originally identified ARID1A binding sites with mouse antibody in the manuscript can be recovered by ARID1A ChIP-seq using rabbit antibody with the same cutoff q < 0.01. The p-value about the significance of such overlaps was less than 0.001. We used the function “fisher” in the bedtools to perform Fisher’s exact test on the number of overlaps [Reference: Aaron R Quinlan, Ira M Hall. BEDTools: A Flexible Suite of Utilities for Comparing Genomic Features. Bioinformatics 26(6), 841-2, 2010].*

*B) From 261 cardiac and 714 neural genes with at least one ARID1A binding peaks in the manuscript, 213 (82% of) cardiac and 631 (88% of) neural genes were found to be associated with ARID1A binding sites confirmed by BOTH mouse and rabbit antibodies. The ratios were higher than all (72%) general ARID1A binding peaks recognized by rabbit antibody as well.*

*C) In Figures 6F and 6I, 10 genes were selected to show the ARID1A binding signals detected by mouse anti-ARID1A antibody. Below, we plotted the ARID1A binding signals on the same genes detected by rabbit anti-ARID1A antibody. Very high consistency can be clearly seen between these two experiments.*

*The ARID1A ChIP-seq using rabbit antibody has been submitted to GEO database and is now pending for the final approval. We will provide the information as soon as we will hear from GEO. As reviewer suggested, we attach a supplemental table including sample information with GSE numbers, alternatively more detailed information can be found from the GEO database directly.*

* Only ARID1A ChIPseq samples are included in GEO GSE139260 submission token. Please include the other genomic datasets (scRNAseq and ATACseq) with this submission. All of the datasets should be deposited in GEO with linked accession numbers and file sets.

*Answer: Indeed, we have submitted scRNA-seq and ATAC-seq to GEO and received individual GSE numbers which are different from the ChIP-seq data. However, we thought all data were linked together with a single token, but it was not. We apologize for the miscommunications. Below are tokens for each individual dataset:*

*GSE139343 elmbgqkitzobnul*

*GSE139342 ejclacaqxbedtyn*

*GSE139329 ongraicsrxgxviz*

*GSE139260 azstasmudhitpyz*

*The ChIP-seq genomic track UCSC in manuscript:*

*https://genome.ucsc.edu/s/samal/hg38_ARID1A_ChIPseq*

*UCSC genome track for ChIP-seq with rabbit antibody:*

*https://genome.ucsc.edu/s/samal/hg38_ARID1A_ChIPseq_rabbit*

*The ChIP-seq data using rabbit ab was uploaded and pending GSE approval.*

* The first sentence of the results section still refers to the "SWI/SNF epigenetic complex." As previously mentioned, please instead refer to it as the SWI/SNF chromatin remodeling complex.

*Answer: Thanks for reviewer’s suggestion. We have changed the phrase to “SWI/SNF chromatin remodeling complex” in the text.*

* As mentioned in the previous review, in Fig. 7h, the input fractions should be probed with the same antibodies used to detect interacting proteins from the pulldowns. The use of GAPDH is not appropriate.

*Answer: Sorry for the confusion. We did not clarify this sufficiently in the manuscript. Indeed, all samples in Figure 7 h-k were collected from the same round of differentiation experiment. At each time point shown in Figure 7h, the cells were evenly separated into two parts. One half was lysed by Complete™ Lysis-M EDTA-free kit (Roche), followed with western-blotting (WB) in Figure 7H. The other half was lysed by Pierce™ Classic Magnetic IP/Co-IP Kit (Thermo Scientific) for CoIP-WB in Figure 7i-k, as well as WB of input again. We did WB for all inputs in Figure 7i-k using the same pull-down antibodies. The reason we did not include them in original version is that we thought those inputs were somehow redundant with Figure 7h. In revised version, we have included all the input WB data again in Figure 7i-k as shown below, which will address the reviewer concern. Thanks a lot for your helpful suggestions.*

* Text legibility has improved for all the figures. However, axes are still not labeled or suitably described in the legend. E.g. Fig. 3c,d,j,k,i. Fig. 3c-d legend states "Violin plots showing expression of..." but does not indicate what these values are. Please be more transparent in the presented values. Are these normalized counts from Seurat? Log or linear? This information is critical to interpretation of the data presented.

*Answer: Thanks to the reviewer for the suggestion. Yes, the values in Violin plots were normalized gene expression generated with the function “LogNormalize” in Seurat. To be more detailed, the gene expression was normalized for each cell by the total expression and multiplied by a scaling factor of 10, 000. The final values were transformed by logarithm scale with the base 10. We have added this information in the Methods and labels “GE (log10)” on y-axis in corresponding plots.*

* Certain statistics are unclear. For example, Fig. 3e-f legend states "* p < 3.13E-163 (Fisher's exact test) (e)", but 6 tests are made. Does that indicate p < 3.13E-163 is the least significant (highest) probability from all 6 tests? Please clarify these points and provide a better description the of tests being performed.

*Answer: Yes. The reviewer’s guess is right. Since the p-values are very significant in our scRNA-seq analysis, it is tedious and become messy/unclear if we list all p-values in the plots. Therefore, we just listed the least statistical significance (the largest p-value) from all tests. We have made clarifications in the text and figure legends.*

* Y-axis scales for genome browser tracks were clarified in the methods section, but these should be added to the respective legends and/or plots for increased legibility while reading. E.g. "Signal is displayed as reads per bp per 10 million reads (RPB10M)."

*Answer: Thanks again for reviewer’s point. We used RPB10M to represent the y-axis scales and gave a clear explanation in the methods section. The y-axis on corresponding figures has been labeled as “RPB10M”.*

Reviewer #3: The authors have addressed most of my concerns and those of the other referees in their response letter and in the revised manuscript. The manuscript has improved somewhat as a result. The experimental evidence for the main conclusions of the study remains somewhat shallow. In fairness it wasn't feasible during a short revision period to obtain additional experimental support for the conclusions as suggested in the first review, and the authors have to some extent toned down their conclusions in the revised version, as requested.

*Answer: Thanks to the reviewer for your recommendation and confirmation of our efforts and improvements in the first revision.*

The quality of the English remains low and the manuscript is still difficult to read. Some of the sentences added to the revised manuscript are hard to understand (example p. 9: "Feature plots from integrative analysis further distinguished distribution patterning of cells, which expressed signature neural and cardiac genes, across all clusters"). The manuscript will need thorough editing before publication.

*Answer: Thanks for reviewer’s suggestion. We invited a native English speaker and senior professor to revise our manuscript again.*

**Third round of review**

The comments to all three reviewers were checked by Referee 2, who had no further concerns.
